# Synthesis and Biological Evaluation of Zeise’s Salt Derivatives with Acetylsalicylic Acid Substructure

**DOI:** 10.3390/ijms19061612

**Published:** 2018-05-30

**Authors:** Alexander Weninger, Daniel Baecker, Victoria Obermoser, Dorothea Egger, Klaus Wurst, Ronald Gust

**Affiliations:** 1 Department of Pharmaceutical Chemistry, Institute of Pharmacy, Center for Molecular Biosciences Innsbruck, University of Innsbruck, CCB—Centrum for Chemistry and Biomedicine, Innrain 80-82, 6020 Innsbruck, Austria; alexander.weninger@uibk.ac.at (A.W.); daniel.baecker@uibk.ac.at (D.B.); victoria.obermoser@gmx.at (V.O.); dorothea.egger@gmx.at (D.E.); 2 Institute of General, Inorganic and Theoretical Chemistry, University of Innsbruck, CCB—Centrum for Chemistry and Biomedicine, Innrain 80-82, 6020 Innsbruck, Austria; klaus.wurst@uibk.ac.at

**Keywords:** anticancer, antiproliferative activity, capillary electrophoresis, COX inhibition, cyclooxygenase enzyme, platinum chemistry, Zeise’s salt

## Abstract

The development of novel biologically active organometallic compounds bearing an acetylsalicylic acid (ASA) substructure led to the synthesis of analogical Zeise-type salts that accordingly inhibit cyclooxygenase (COX) enzymes. In order to determine the influence of the length of the alkyl chain between the platinum(II) center and the ASA moiety, compounds with varying methylene groups (*n* = 1–4) were synthesized and characterized. For the propene derivative structural elucidation by X-ray crystallography was possible. Prior to evaluation of biological activity, the complexes were investigated regarding their stability in different media, such as water, physiological sodium chloride, and phosphate buffered saline. Therefore, an analytical method based on capillary electrophoresis was established. All of the compounds were tested for their COX inhibitory potential. In general, complexes with longer alkyl chains caused higher inhibition of COX enzymes and the inhibitory potential towards COX enzymes was enhanced when compared to Zeise’s salt. The growth inhibitory effects of the synthesized substances were investigated in vitro against colon carcinoma (HT-29) and breast cancer (MCF-7) cells. The IC_50_ values of the new derivatives ranged from 30 to 50 µM, whereas neither Zeise’s salt itself nor ASA showed any antiproliferative activity at the used concentrations.

## 1. Introduction

**Figure 1:**
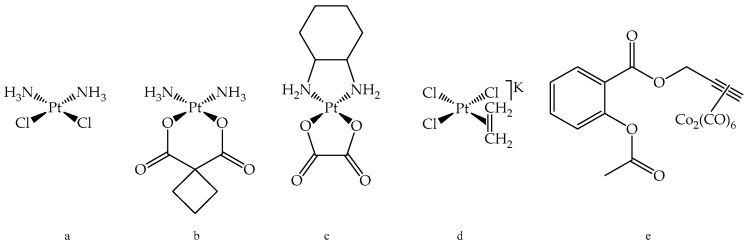
(**a**) Cisplatin; (**b**) Carboplatin; (**c**) Oxaliplatin; (**d**) Zeise’s salt; and (**e**) Co-ASS.

The most famous platinum complex in anticancer therapy by far is Cisplatin, which was first synthesized by M. Peyrone in 1844 [1]. However, it took more than 120 years until its antitumor effect was discovered by B. Rosenberg [2], and to date it is in therapeutic use for 40 years. During that time, various derivatives of Cisplatin were synthesized that differ in the leaving groups and/or in the carrier ligands to improve its pharmacological profile. Yet, only a few derivatives, such as Carboplatin and Oxaliplatin, are approved for tumor therapy [3,4,5]. Meanwhile, the DNA was identified to be the main target of platinum complexes where intrastrand cross-links between adjacent guanine bases or adjacent guanine and adenine bases are formed, causing defective replication mechanisms and ultimately leading to cell death [4]. Cisplatin, Carboplatin, and Oxaliplatin (Figure 1) have in common that they are restricted by dose limiting side effects as well as frequent development of resistance. Unfortunately, they show cross-resistance due to the same mode of action by targeting the DNA [4,5].

To overcome these disadvantages and to obtain alternative drugs for a second line therapy, novel approaches are aimed at following the use of other metal centers or the design of bioorganometallic compounds, which address other targets than the DNA [3,6,7,8,9,10,11,12,13,14,15,16,17].

A possible target is represented by both cyclooxygenase (COX) enzymes. The COX-1 isoform is constitutively active in nearly all human tissues, whereas the isoenzyme COX-2 is basally expressed only in the central nervous system, the kidney and in female reproduction organs [18]. Relevant upregulation of COX-2 takes place during inflammation or carcinogenesis. Overexpression of COX-2 in various types of tumors, such as prostate, colon, or breast cancer, is also well known. Moreover, the prognosis is in general poor for patients suffering breast and prostate carcinoma, which display a high COX expression [19,20]. It was shown in animal models that the use of COX-inhibitors led to the reduction of both growth and progression of these tumors. Such inhibitors are renowned as nonsteroidal anti-inflammatory drugs (NSAIDs), of which acetylsalicylic acid (ASA) might be the most famous representative.

**Scheme 1:**
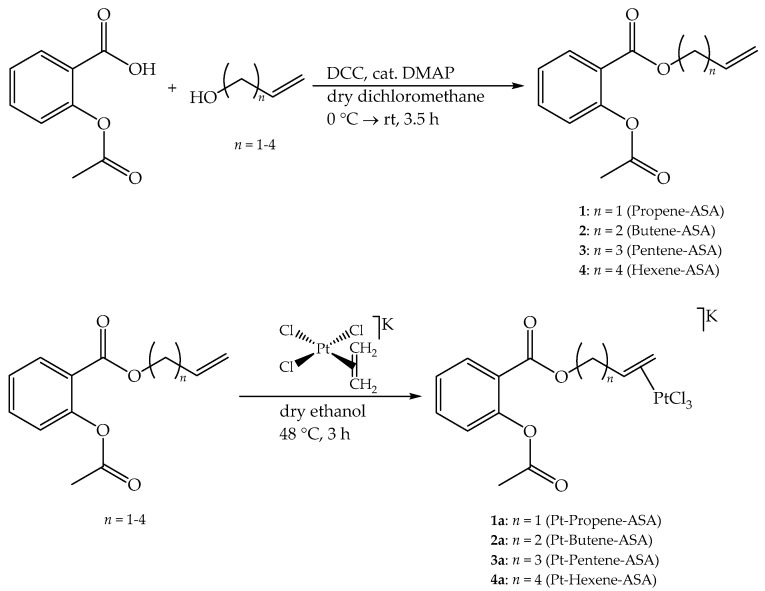
Syntheses of the ligands (top row) and of the final Zeise’s salt derivatives (bottom row). All of the reactions were carried out under a protective atmosphere of argon. rt = room temperature.

A series of metal complexes bearing the ASA substructure was already investigated by our group [12,13,14,15]. In this context, ASA derivatives of Zeise’s salt were examined as well [21]. Zeise’s salt, potassium trichlorido[ethylene]platinate(II) (see Figure 1), was named after its discoverer, the Danish pharmacist W. C. Zeise. This compound is considered as one of the first, if not the very first, organometallic compound. In contrast to Cisplatin, Zeise’s salt was never under clinical investigation, because of its high instability. However, we demonstrated that Zeise’s salt is pharmacologically active as it inhibited the COX isoenzymes during an incubation time of 10 min [21]. Binding of Zeise’s salt via a methylene spacer to the carboxylic group of ASA analogously to the design of Co-ASS (see Figure 1) increased the stability, as well as the COX inhibitory potency. The resulting Pt-Propene-ASA complex (**1a**, see Scheme 1) represents not only a COX-1/2 inhibitor, but it also suppressed the growth of various tumor cells [21].

Interestingly, if the degradation of Pt-Propene-ASA takes place, the Zeise moiety is not reduced but a platinum mediated ester cleavage takes place.

These findings unequivocally showed that Zeise’s salt derivatives represent a promising class of organometallic compounds to be optimized as antitumor agents with a mode of action different from already established platinum complexes. However, it is necessary to further improve the stability in aqueous solution in order to realize preclinical evaluations.

In this study, the alkyl spacer between ASA and the Zeise component was elongated in **1a** (–(CH_2_)*_n_*– with *n* = 1–4) in order to reduce the influence of the platinum on the ester bonding. The homologous series of Pt-Propene-ASA (*n* = 1: **1a**), Pt-Butene-ASA (*n* = 2: **2a**), Pt-Pentene-ASA (*n* = 3: **3a**), and Pt-Hexene-ASA (*n* = 4: **4a**) was investigated regarding the COX inhibitory potential and the influence on the proliferation of the HT-29 colon carcinoma and the MCF-7 breast cancer cell lines.

## 2. Results

### 2.1. Syntheses and Characterization

#### 2.1.1. Syntheses

The syntheses of the target compounds **1a**–**4a** are summarized in Scheme 1. The ligands **1**–**4** were obtained by conventional Steglich esterification of ASA with the respective alkenole [22]. In a first step, ASA built with *N,N*′-dicyclohexylcarbodiimide (DCC) in the presence of catalytic amounts of 4-dimethylaminopyridine (DMAP) in dry dichloromethane an active *N*-acyl species that readily reacted at a low temperature (0 °C → room temperature (rt)) in a second step with the respective alcohol. Complexation of the resulting esters **1**–**4** with Zeise’s salt was possible in dry ethanol by stirring at elevated temperature within 3 h in a slight excess of the ligand. All of the desired products were obtained in overall good yields. It is notable that the final organometallic compounds are hygroscopic and sensitive to light. Thus, they should be stored in a desiccator with protection from light.

The platinum compounds are easily soluble in polar solvents, such as water, alcohol, and acetone, and they are insoluble in ether or hydrocarbons. In dimethyl sulfoxide (DMSO) rapid Cl^−^/DMSO exchange took place, as confirmed by NMR spectroscopy.

#### 2.1.2. X-ray Structure Analysis

**Figure 2:**
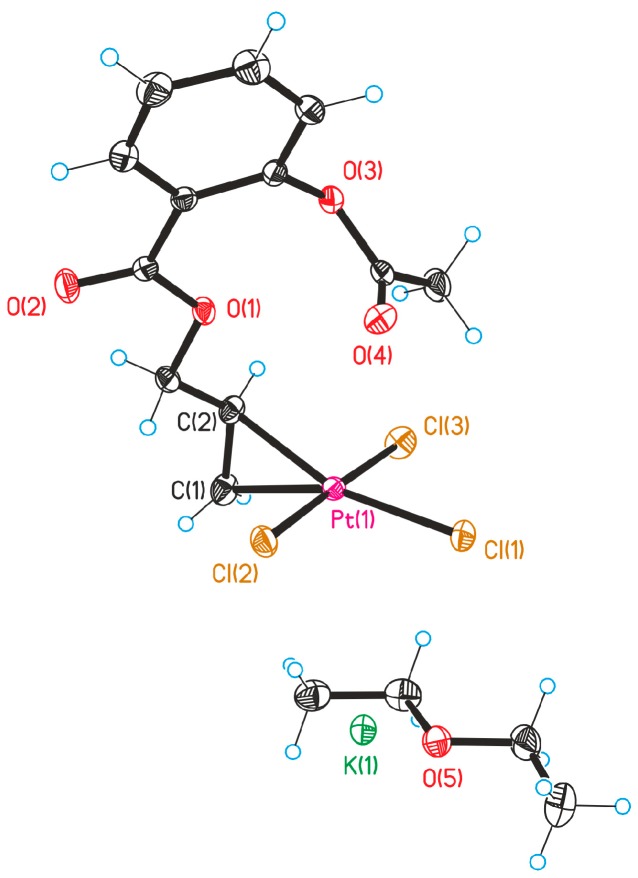
ORTEP drawing of complex **1a**.

The structure of the Zeise’s salt derivatives was determined by X-ray analysis on the example of Pt-Propene-ASA (**1a**). Crystal data and refinement for **1a** are submitted in the Supplementary Materials. Selected bond lengths, distances, and angles are listed in Table 1. For the numbering of the atoms, see Figure 2.

**Figure 3:**
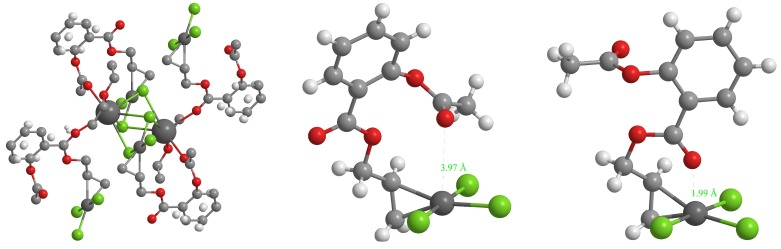
Left: K^+^ interactions in the crystal of **1a**; middle: single molecule of **1a**; right: conformational change of the ASA moiety in **1a**.

Crystals of **1a** were obtained from a mixture of ethanol/diethyl ether. In the crystal, the potassium ion is fixed by interactions to two carbonyl oxygens, the oxygen of the co-crystallized ether molecule and the four chlorido ligands of two PtCl_3_ moieties (Figure 3, left). The olefin is perpendicularly π-bonded at the PtCl_3_ moiety in a square planar coordination sphere around the platinum (see Figure 2 and Figure 3, middle).

The distance from the midpoint of the C-C double bond of **1a** to the platinum centre (2.015 Å) is slightly smaller, as in Zeise’s salt (2.022 Å) [23]. The dπ-pπ* back bonding to the vinyl group is stronger in **1a** because of the electron drawing properties of the ASA-CH_2_- residue and slightly reduces the length of the C-C bond (1.40 Å) when compared to that of Zeise’s salt (1.44 Å). Contrary to expectations, the Pt-Cl bond oriented *trans* to the ethylene is not substantially elongated (2.32 Å) compared to the other ones (2.30 Å), which points to only a marginal *trans* effect.

Overall, the trichlorido[ethylene]platinate moiety of **1a** is very similar to Zeise’s salt regarding bond lengths and angles. The ASA moiety is oriented in the crystal above the square planar coordination plane. Thereby, the carbonyl oxygen of the acetoxy moiety is at a distance of 3.97 Å above the platinum (Figure 3, middle).

#### 2.1.3. Molecular Characterization by NMR Spectroscopy

All of the synthesized ligands and the corresponding complexes were fully characterized by ^1^H and ^13^C NMR spectroscopy. The assignment of the signals was performed by [^1^H,^1^H]-COSY, [^1^H,^13^C]-HSQC, and [^1^H,^13^C]-HMBC 2D NMR experiments (see Supplementary Materials).

The coordination of the ligands to platinum was confirmed by a significant upfield shift of the vinyl protons. The alkene is bound to the platinum in a π-donor/π-acceptor bonding, thus reducing the double bond character (to about 1.5 as determined in the X-ray structure). As a consequence, the resonances of the vinyl protons and the carbons of the platinum complexes are shifted 0.87–1.06 and 49.83–55.87 ppm, respectively, to higher field (summarized in Table 2).

**Figure 4:**
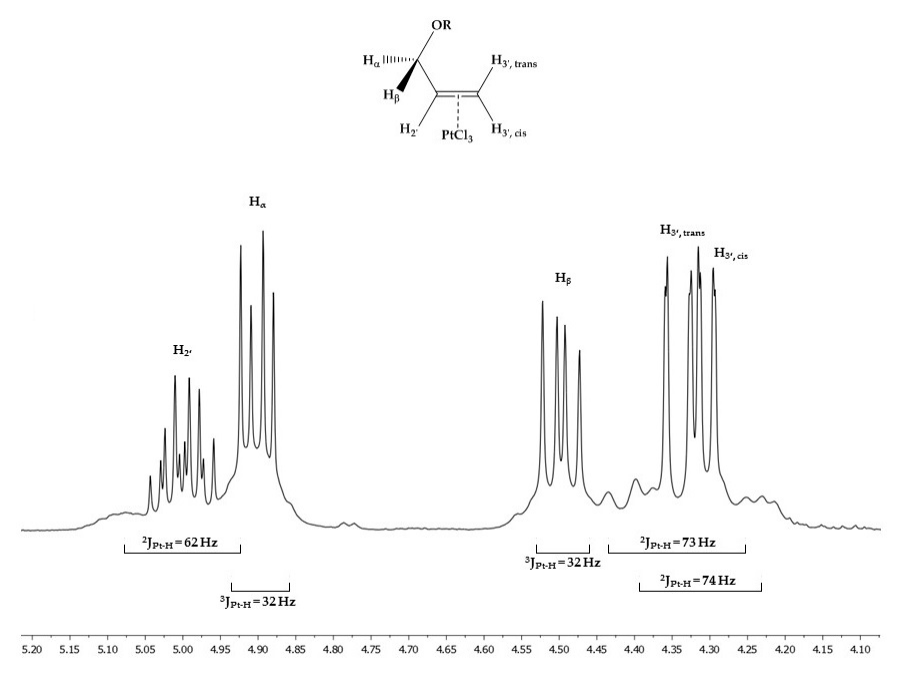
Excerpt of the ^1^H NMR spectrum of **1a** featuring the ^2/3^J_Pt–H_ couplings and the assignments of the proton signals. The methylene protons show a diastereotopic splitting.

Because of the unsymmetrical substitution, three =C-H resonances appear in the ^1^H NMR spectra, splitted by geminal and vicinal couplings. When compared to Zeise’s salt (δ = 4.20), they are shifted to lower field (e.g., δ (**1a**) = 4.31, 4.34 (=CH_2_) and 5.00 (=CH–R)). The ^2^J_Pt-H_ remained nearly unchanged (Zeise’s salt: 64 Hz; **1a**: 73 Hz (=CH_2_) and 62 Hz (=CH–R), see Figure 4).

Interestingly, the ^1^H NMR spectra demonstrate a diastereotopic splitting of the –CH_2_–CH= methylene protons for the complexes **1a**–**4a**. In the spectrum of **1a** (see Figure 4), the resonances are at δ = 4.90 (dd, ^2^J = 11.9 Hz, ^3^J = 5.5 Hz, 1H, –OC**H_α_**H_β_–), and 4.50 (dd, ^2^J = 11.9 Hz, ^3^J = 7.7 Hz, 1H, –OCH_α_**H_β_**–). The ^3^J couplings of the –CH_2_– protons to –CH= reveal a conformational change of the ASA-CH_2_-moiety. When compared to the dihedral angles found in the X-ray structure (60° and 180°), the coupling constants of 5.5 Hz and 7.7 Hz point to a reduction of the dihedral angle to 40–50° and 140–150°, respectively. The ^3^J_Pt-H_ couplings of about 32 Hz confirm this change (see Figure 4).

While in the crystal of **1a**, the spatial structure of the ligand is determined by interactions to potassium, it can adopt an energetically more preferred conformation in solution. This can include a reorientation of the carbonyl oxygen of the ester above the platinum (see Figure 3, right). In this case, a Pt-O distance of about 2 Å can be realized, which is very similar to those of platinum(IV) acetate complexes [24]. This reorientation facilitates the nucleophilic attack of a water molecule at the carbonyl C-atom, because the transition state is stabilized by the Pt-O-C interaction. When compared to a “normal” ester function the attack of a water molecule is eased and the ester is cleaved platinum-mediated.

#### 2.1.4. Characterization by Mass Spectrometry

Platinum has six naturally occurring isotopes (^190^Pt, ^192^Pt, ^194^Pt, ^195^Pt, ^196^Pt, ^198^Pt), of which five are stable (not stable: ^190^Pt). Upon complexation characteristic mol peaks with the significant line pattern representing these platinum isotopes confirmed the binding of the ethylene moiety to the platinum. As an example, the mass spectrum of **1a** is submitted in the Supplementary Materials.

### 2.2. Evaluation of Stability

Capillary electrophoresis (CE) was employed in order to determine the stability of the anionic platinum(II) complexes in aqueous media. Therefore, a method was established that allowed the detection of the respective complex and possible degradation products in a short period of time (for further information on the setup see Section 4). Methanol was chosen as a non-coordinating co-solvent for the sample solutions (final amount of methanol: 50%) in order to guarantee the solubility of decomposition products. In contrast to HPLC analysis, it was possible to separate the platinum complex (*t*_m_ = 6.3 min) from the free ligand (*t*_m_ = 4.4 min) using a 50 mM sodium tetraborate solution (pH = 9.3) as the background electrolyte (temperature: 25 °C; UV-detection: 230 nm; capillary: fused silica 64.5 cm × 75 µm, effective length 56 cm; 20 kV). The possible degradation products ASA and salicylic acid (SA) showed migration times of 7.0 min and 8.2 min, respectively. The reactions followed pseudo-first order kinetics (*R*^2^ ≥ 0.99) under the experimental conditions that were implemented.

Complex **1a** was stable in methanol for 48 h. Upon the addition of water (1:1 mixture), a fast decomposition of **1a** took place. As previously determined by HPLC analysis, the main degradation was the cleavage of ASA from the [propenol]PtCl_3_ moiety. In a further step, ASA was deacetylated to SA. Neither a release of the ligand **1** from the platinum nor oxidation reactions of Pt(II) were detected. The half-live (τ_1/2_) of **1a** was determined to be 35.7 ± 0.6 min. This value is in good agreement with the result published before [21].

The butenol derivative **2a** was much more stable with τ_1/2_ = 69.6 ± 3.0 h, indicating that an additional methylene group between ASA and the alkene-platinum reduced the influence of the platinum on the ester cleavage. While in **1a** the carbonyl group is arranged to the platinum in a six-membered ring, the additional methylene group would enlarge the ring, for which reason other conformational orientations might be energetically preferred. A platinum-mediated ester cleavage played a subordinate role and the stability increased. Further elongation of the spacer between ASA and the Zeise moiety did not further improve the stability (τ_1/2_ = 73.5 ± 4.7 h for **3a** and τ_1/2_ = 55.4 ± 4.0 h for **4a**).

Substitution reactions with chloride ions can take place under physiological conditions [25]. For instance, high Cl^−^ concentrations can lead to a substitution of the alkene ligand and the formation of the tetrachloridoplatinate(II) complex. Therefore, the stability of **2a** was examined in the presence of 154 mM Cl^−^ (extracellular Cl^−^ concentration) in the 50% methanol/water mixture. Interestingly, instead of the alkene release, the stability significantly increased (recovery of 80.3 ± 3.2% of the intact complex **2a** after 168 h). This finding points to the hindered positioning of the carbonyl oxygen above the platinum in the presence of high chloride concentrations.

The stability of **2a** was further investigated in the presence of phosphate-buffered saline (PBS). In PBS, the chloride concentration is comparable to that used in the above described experiment, but it is additionally buffered with potassium phosphate to a physiological pH of 7.4. The same conditions were adjusted in the methanol/water mixture.

To our great surprise, incubation of **2a** with PBS resulted in a faster degradation (τ_1/2_ = 7.95 ± 0.09 h) as in methanol/water. However, no cleavage of the alkenol ester took place, but **2a** was degraded to Pt-Butene-SA. The release of the acetate results in an organometallic compound with lower mobility and a characteristically changed UV-spectrum. This reaction happened most probably due to the slightly basic conditions in PBS, allowing for alkaline-caused hydrolysis of the acetyl ester group.

Nevertheless, these results clearly demonstrated that residues at the ethylene ligand of Zeise’s salt have an impact on the stability of the complex. Zeise’s salt itself is very unstable under aqueous conditions [25] and it degrades in a redox reaction building acetaldehyde and Pt(0). This reaction was not observed in case of the complexes **1a**–**4a**. To determine the biological effects of Zeise’s salt only short time incubations (up to 15 min) are possible, e.g., determination of COX the inhibition (see Section 2.3.1; incubation time: 10 min). Other experiments, such as cytotoxicity assays (see Section 2.3.2; incubation time 72 h), are only significant for the complexes **1a**–**4a**.

### 2.3. Biological Evaluation

#### 2.3.1. COX-1/2 Isoenzyme Inhibition

**Figure 5:**
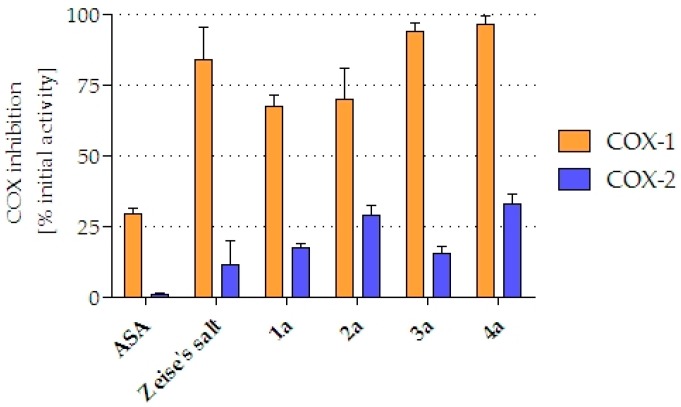
Inhibition of isolated ovine COX-1 and COX-2. Values are given as % COX inhibition calculated from initial activity of vehicle treated enzyme and represent the mean ± standard deviation of ≥2 independent experiments.

The impact of the compounds (10 µM) to inhibit the isoenzymes COX-1 and COX-2, respectively, was investigated (Figure 5). The parent compound ASA displayed a COX-1 inhibition of 29.2% and was inactive towards COX-2 (1.0%). In contrast, Zeise’s salt and its derivatives revealed greater inhibitory properties. Zeise’s salt caused a strong inhibition of COX-1 (83.8%), while it showed just a marginal reduction of COX-2 activity (11.3%). The inhibitory potency of the complexes **1a**–**4a** depended on the spacer between ASA and the Zeise moiety. The compounds **1a** and **2a** exhibited slightly less COX-1 inhibition when compared to Zeise’s salt (67.3% and 70.0%, respectively). **3a** (93.8%) and **4a** (96.6%) nearly reached a 100% inhibition. The same trend was observed at COX-2, however, on a much lower level: Zeise’s salt (11.3%) < **1a** (17.4%) < **2a** (28.7%) < **4a** (33.0%). Only **3a** did not fit this series. It reduced COX-2 merely to the same amount (15.3%) as Zeise’s salt.

#### 2.3.2. Antiproliferative Effects

The growth inhibitory effects of ASA, Zeise’s salt as well as the thereof derived complexes **1a**–**4a** were investigated in vitro against the HT-29 and the MCF-7 cell lines. Thereby, an already published crystal violet assay [26] was employed, in which the cell biomass reduced by the substances was determined as parameter. Cisplatin was applied as positive control. The resulting IC_50_ values are listed in Table 3.

ASA and Zeise’s salt did not show antiproliferative activity towards both cell lines at the used concentrations (IC_50_ ≥ 50 µM). Both of the cell lines are comparably sensitive to the Zeise’s salt derivatives. The lowest effects showed **1a** with a 50% inhibition at 50 µM (IC_50_ ≈ 50 µM). Elongation of the spacer by one methylene group (**2a**) increased the cytotoxicity to an IC_50_ value of about 31 µM. Further elongation slightly reduced the activity, but **3a** (IC_50_ (HT-29) = 44.2 µM; IC_50_ (MCF-7) = 37.4 µM) and **4a** (IC_50_ (HT-29) = 41.4 µM; IC_50_ (MCF-7) = 43.7 µM) were even more active than ASA and Zeise’s salt. However, all of the complexes were 10- to 20-fold less active than Cisplatin (IC_50_ (HT-29) = 2.6 µM and IC_50_ (MCF-7) = 3.7 µM; see Table 3).

## 3. Discussion

NSAIDs are drugs with analgetic, antiphlogistic, anti-inflammatory, and anticoagulative properties. All of them share the ability to inhibit cyclooxygenase enzymes.

Out of this drug class ASA is the most prominent representative and is the subject of extensive pharmacological investigations. During the last years, various epidemiological studies revealed that ASA could be used for the prevention of tumor development, such as colon and breast cancer [27,28]. However, no information is available that ASA can be used for the treatment of established tumors. Nevertheless, COX enzymes might be targets for the design of new anticancer drugs, because some tumor entities grow under the influence of COX.

In our studies, ASA was inactive against the HT-29 colon cancer and the MCF-7 breast cancer cell lines in vitro up to a concentration of 100 µM. In contrast, Zeise’s salt derivatives, which are more potent inhibitors of COX-1/2, caused antiproliferative effects against these cell lines. Zeise’s salt itself is not stable in cell culture medium and it degraded immediately after addition. The derivatives **1a**–**4a** might also be not stable under these conditions, but as the experiments with **2a** in PBS demonstrated, at first, only a loss of the acetyl residue can be assumed preventing the essential trichlorido[alkene]platinum(II) moiety. The high chloride concentration should decrease the cleavage of ASA or SA from the trichlorido[alkenol]platinum(II) moiety.

However, degradation by ester cleavage might be the most critical point. Complex **1a** having the lowest stability under aqueous conditions showed the lowest activity against the used cell lines. The derivatives **2a**–**4a**, which were more stable, reduced the cell growth with IC_50_ values that were between 30 and 40 µM. When compared to Cisplatin (IC_50_ values of about 3 µM), they are more than 10-fold less active.

The reason for this weaker cytotoxicity might be the difference of the intracellular targets. While the main target of Cisplatin represents the DNA, Zeise’s salt derivatives can bind to isolated DNA [21], but it seems that the intrastrand cross-links, which are necessary for high cytotoxicity, are not built in the case of Zeise’s salt derivatives or were not as stable as found for Cisplatin.

As already published earlier, Cisplatin and Zeise’s salt showed nearly the same uptake kinetic in tumor cells, while **2a** was four- to five-fold higher accumulated. Therefore, partial inactivation by ingredients of the cell culture medium can also be considered as a reason for their low cytotoxicity.

Although the X-ray structure revealed only a marginal elongation of the Pt–Cl bond *trans* to the alkene, it is well accepted that the *trans* effect of the alkene moiety forces on the one hand a fast exchange of the chlorido ligand *trans* to the olefin by stronger ligands, such as amines and thiols. On the other hand strong σ-donors might have a weakening effect on the Pt-alkene bonding and can cause olefin release. Incubation of **2a** with an excess of l-cysteine yielded a yellow precipitate that was not soluble in common solvents. We assume that Pt(Cys)_2_ was built, which is a well-known degradation product of Cisplatin. Formation of amino acid-platinum compounds is likewise responsible for the partial deactivation of Cisplatin upon injection into the patient’s blood stream [29,30].

In summary, we synthesized a series of Zeise’s salt derivatives with ASA substructure. We were able to enhance the stability of the complexes on the one hand and the COX inhibitory potential on the other. Interference in the arachidonic acid cascade as mode of action is assumed. However, further investigations regarding the mode of action are necessary and the improvement of activity at the COX enzymes is currently undertaken.

## 4. Materials and Methods

### 4.1. General Aspects

All of the chemicals were purchased either from Sigma-Aldrich, Alfa Aesar, Fluka, Euriso-Top, or TCI Chemicals and were used as received without further purification. Solvents were purchased in appropriate purity or were freshly distilled prior to utilization. Water was deionized using a Millipore Milli-Q Gradient A10 Water Purification system (Merck Millipore, Billerica, MA, USA). Column chromatography was performed using silica gel 60 (particle size 40–63 µm). Reactions were monitored by thin layer chromatography (TLC) that was carried out on “TLC Silica gel 60 F254 aluminium sheets” with a fluorescence indicator (Merck, Darmstadt, Germany). Detection was performed by observation under UV-light at 254 and/or 365 nm, respectively. Melting points (m.p.) were determined using a Kofler hot bench (Wagner & Munz, Munich, Germany) and uncorrected values are given.

^1^H and ^13^C NMR spectroscopy was performed employing either a Bruker Avance 4 Neo (^1^H resonance frequency: 400 MHz) or an Agilent Direct Drive 2 (^1^H resonance frequency: 500 MHz) spectrometer. For the correct assignment of the signals, [^1^H,^1^H]-COSY, [^1^H,^13^C]-HSQC and [^1^H,^13^C]-HMBC 2D NMR experiments were carried out. Chemical shifts δ are given in parts per million (ppm). Coupling constants J are given in Hertz (Hz). Chemical shifts of ^1^H and ^13^C experiments were referenced using the center of the internal residual peak of the solvent multiplet, which was related to tetramethylsilane (TMS) as δ = 2.05 (^1^H NMR) and δ = 29.84 (^13^C NMR) for Acetone-*d6* [31]. The subscripts α and β indicate the downfield shifted proton (subscript α), respectively, upfield shifted proton (subscript β) of a diastereotopic methylene moiety.

High-resolution electrospray ionization mass spectrometry (HR-ESI-MS) was performed using an Orbitrap Elite mass spectrometer (Thermo Fisher Scientific, Waltham, MA, USA). The ligands were analyzed in positive mode and the platinum complexes in negative mode. The peaks of greatest intensity are presented.

Capillary electrophoresis experiments were carried out on a 3D-CE system (Agilent, Santa Clara, CA, USA), which was equipped with an autosampler, a diode array detector (DAD) and a temperature controlled column compartment. Agilent fused-silica capillaries (75 µm i.d.; 56 cm effective length, 64.5 cm total length) were purchased from VWR (Vienna, Austria).

The purity of all the compounds was above 95%, as determined by CE.

### 4.2. Capillary Electrophoresis

A 50 mM sodium tetraborate solution with a pH of 9.3 (adjusted with 1 M NaOH solution) was used as the background electrolyte (BGE). Prior to the first utilization new capillaries were washed with 1 M NaOH solution (45 min), water (45 min) and BGE (45 min). Samples were injected into the capillary via hydrodynamic mode (50 mbar for 2 s). The capillary was thermostated at 25 °C, whereas the autosampler was kept at 37 °C by an external water bath. The separation voltage was 20 kV and the detection wavelength was set to 230 nm. The required run time was 10 min. The capillary was flushed consecutively with 0.1 M NaOH solution (3 min), water (3 min), and BGE (5 min) before each measurement. For the purpose of stability testing, the complexes were dissolved in methanol and diluted 1:1 (*v*/*v*) with water, 1.8% aqueous NaCl solution, or double-concentrated PBS to a final concentration of 1 mM. Every experiment was performed in triplicate. The half-lives are presented as mean ± standard deviation. All of the samples, buffers, and washing solutions were membrane filtered (0.22 µm porosity) and degassed by ultrasonication prior to usage.

### 4.3. X-ray Crystallography

For single crystal structure analysis, a suitable crystal has been prepared under a polarization microscope and immediately placed into a stream of cold nitrogen (173 K) inside a Bruker D8 Quest diffractometer (Photon 100) that was equipped with an Incoatec Microfocus source generator (multi layered optics monochromatized Mo-*K_α_* radiation, *λ* = 71.073 pm). Multi-scan absorption corrections were applied with the program SADABS-2014/5. After structure solution and parameter refinement with anisotropic displacement parameters for all of the atoms using the SHELXS/L-13 [32,33] software suite, the space group *P2*_1_/*c* was found to be correct. Hydrogen atoms at C1 and C2 were found and refined with isotropic displacement parameters without any restraints. Additional details of the crystal structure investigation may be obtained from the Cambridge Crystallographic Data Centre (CCDC). The supplementary crystallographic data of **1a** were deposited as CCDC number 1831275 and these data are provided free of charge.

### 4.4. General Procedure for the Synthesis of the Acetylsalicylic Acid Esters ***1***–***4***

Following the protocol of Steglich et al. [22], under a protective atmosphere of argon 1.00 equivalents of ASA (10.0 mmol), 1.10 equivalents of the respective alcohol (11.0 mmol) and 10.0 mol% DMAP were dissolved in 30 mL of anhydrous dichloromethane and were cooled with an ice bath. Then, a solution containing 1.05 equivalents of DCC (10.5 mmol) in 10 mL of anhydrous dichloromethane was added over a period of 5 min via syringe. The reaction mixture was stirred for another 10 min at 0 °C, and then for 3.5 h at room temperature. After completion of the reaction (TLC monitoring; eluent: petroleum ether/ethyl acetate = 5:1), the mixture was washed each three times with aqueous 1 M HCl solution and saturated aqueous NaHCO_3_ solution and once with brine. The organic phase was dried over anhydrous Na_2_SO_4_ and evaporated. Pure products were obtained after column chromatography (petroleum ether/ethyl acetate = 5:1).

#### 4.4.1. (Prop-2-en-1-yl)-2-acetoxybenzoate (Propene-ASA, **1**)

Yield: 80% as colorless oil. HR-ESI-MS: calculated for C_12_H_12_O_4_Na [**1**+Na]^+^: 243.0628. Found: *m*/*z* 243.0628. ^1^H NMR (400 MHz, Acetone-*d6*): δ = 8.02 (dd, ^3^J = 7.8 Hz, ^4^J = 1.7 Hz, 1H, ArH-6), 7.66 (ddd, ^3^J = 8.1 Hz, ^3^J = 7.5 Hz, ^4^J = 1.7 Hz, 1H, ArH-4), 7.40 (ddd, ^3^J = 7.7 Hz, ^3^J = 7.7 Hz, ^4^J = 1.2 Hz, 1H, ArH-5), 7.20 (dd, ^3^J = 8.1 Hz, ^4^J = 1.2 Hz, 1H, ArH-3), 6.06 (ddt, ^3^J = 17.3 Hz, ^3^J = 10.5 Hz, ^3^J = 5.7 Hz, 1H, –C**H**=CH_2_), 5.42 (ddt, ^3^J = 17.2 Hz, ^2^J = ^4^J = 1.6 Hz, 1H, =CH_2,_ *_trans_*), 5.28 (ddt, ^3^J = 10.5 Hz, ^2^J = ^4^J = 1.4 Hz, 1H, =CH_2,_ *_cis_*), 4.78 (dt, ^3^J = 5.7 Hz, ^4^J = 1.4 Hz, ^4^J = 1.4 Hz, 2H, –OCH_2_–), 2.26 (s, 3H, –CH_3_); ^13^C NMR (101 MHz, Acetone-*d6*): δ = 169.69 (–(**C**=O)–CH_3_), 164.76 (Ar-(**C**=O)), 151.72 (C2), 134.83 (C4), 133.43 (C2′), 132.27 (C6), 126.86 (C5), 124.92 (C3), 124.59 (C1), 118.65 (C3′), 66.23 (C1′), 21.01 (–CH_3_).

#### 4.4.2. (But-3-en-1-yl)-2-acetoxybenzoate (Butene-ASA, **2**)

Yield: 78% as colorless oil. HR-ESI-MS: calculated for C_13_H_14_O_4_Na [**2**+Na]^+^: 257.0784. Found: *m*/*z* 257.0785. ^1^H NMR (400 MHz, Acetone-*d6*): δ = 8.00 (dd, ^3^J = 7.8 Hz, ^4^J = 1.8 Hz, 1H, ArH-6), 7.65 (ddd, ^3^J = 8.1 Hz, ^3^J = 7.4 Hz, ^4^J = 1.7 Hz, 1H, ArH-4), 7.38 (ddd, ^3^J = 7.6 Hz, ^3^J = 7.6 Hz, ^4^J = 1.2 Hz, 1H, ArH-5), 7.19 (dd, ^3^J = 8.1 Hz, ^4^J = 1.2 Hz, 1H, ArH-3), 5.90 (ddt, ^3^J = 17.1 Hz, ^3^J = 10.3 Hz, ^3^J = 6.8 Hz, 1H, –C**H**=CH_2_), 5.18 (ddt, ^3^J = 17.2 Hz, ^2^J = ^4^J = 1.7 Hz, 1H, =CH_2,_ *_trans_*), 5.08 (ddt, ^3^J = 10.3 Hz, ^2^J = 2.3 Hz, ^4^J = 1.3 Hz, 1H, =CH_2,_ *_cis_*), 4.32 (t, ^3^J = 6.7 Hz, 2H, –OCH_2_–), 2.51 (tdt, ^3^J = 6.7 Hz, ^3^J = 6.7 Hz, ^4^J = 1.4 Hz, 2H, –CH_2_–), 2.28 (s, 3H, –CH_3_); ^13^C NMR (101 MHz, Acetone-*d6*): δ = 169.65 (–(**C**=O)–CH_3_), 164.97 (Ar-(**C**=O)), 151.73 (C2), 135.26 (C3′), 134.71 (C4), 132.20 (C6), 126.79 (C5), 124.89 (C3), 124.67 (C1), 117.59 (C4′), 64.76 (C1′), 33.79 (C2′), 21.02 (–CH_3_).

#### 4.4.3. (Pent-4-en-1-yl)-2-acetoxybenzoate (Pentene-ASA, **3**)

Yield: 60% as colorless oil. HR-ESI-MS: calculated for C_14_H_16_O_4_Na [**3**+Na]^+^: 271.0941. Found: *m*/*z* 271.0920. ^1^H NMR (500 MHz, Acetone-*d6*): δ = 8.02 (dd, ^3^J = 7.8 Hz, ^4^J = 1.5 Hz, 1H, ArH-6), 7.65 (ddd, ^3^J = 8.1 Hz, ^3^J = 7.3 Hz, ^4^J = 1.6 Hz, 1H, ArH-4), 7.39 (ddd, ^3^J = 7.6 Hz, ^3^J = 7.6 Hz, ^4^J = 1.1 Hz, 1H, ArH-5), 7.19 (d, ^3^J = 8.1 Hz, 1H, ArH-3), 5.88 (ddt, ^3^J = 17.0 Hz, ^3^J = 10.2 Hz, ^3^J = 6.7 Hz, 1H, –C**H**=CH_2_), 5.08 (ddt, ^3^J = 17.2 Hz, ^2^J = ^4^J = 1.6 Hz, 1H, =CH_2,_ *_trans_*), 4.99 (ddt, ^3^J = 10.2 Hz, ^2^J = 2.2 Hz, ^4^J = 1.2 Hz, 1H, =CH_2,_ *_cis_*), 4.28 (t, ^3^J = 6.6 Hz, 2H, –OCH_2_–), 2.28 (s, 3H, –CH_3_), 2.24–2.18 (m, 2H, –C**H**_2_–CH=), 1.85 (tt, ^3^J = 7.0 Hz, ^3^J = 7.0 Hz, 2H, –CH_2_–); ^13^C NMR (126 MHz, Acetone-*d6*): δ = 169.63 (–(**C**=O)–CH_3_), 165.01 (Ar-(**C**=O)), 151.67 (C2), 138.59 (C4′), 134.65 (C4), 132.20 (C6), 126.79 (C5), 124.85 (C3), 124.69 (C1), 115.59 (C5′), 65.08 (C1′), 30.76 (C3′), 28.63 (C2′), 21.01 (–CH_3_).

#### 4.4.4. (Hex-5-en-1-yl)-2-acetoxybenzoate (Hexene-ASA, **4**)

Yield: 82% as colorless oil. HR-ESI-MS: calculated for C_15_H_18_O_4_Na [**4**+Na]^+^: 285.1097. Found: *m*/*z* 285.1070. ^1^H NMR (500 MHz, Acetone-*d6*): δ = 8.00 (dd, ^3^J = 7.9 Hz, ^4^J = 1.7 Hz, 1H, ArH-6), 7.67–7.62 (m, 1H, ArH-4), 7.39 (dd, ^3^J = 7.6 Hz, ^3^J = 7.6 Hz, 1H, ArH-5), 7.19 (d, ^3^J = 7.9 Hz, 1H, ArH-3), 5.84 (ddt, ^3^J = 17.0 Hz, ^3^J = 10.1 Hz, ^3^J = 6.7 Hz, 1H, –C**H**=CH_2_), 5.08–5.00 (m, 1H, =CH_2,_ *_trans_*), 4.95 (ddt, ^3^J = 10.2 Hz, ^2^J = 2.1 Hz, ^4^J = 1.0 Hz, 1H, =CH_2,_ *_cis_*), 4.28 (t, ^3^J = 6.7 Hz, 2H, –OCH_2_–), 2.28 (s, 3H, –CH_3_), 2.13 (dt, ^3^J = 7.8 Hz, ^3^J = 7.8 Hz, 2H, –C**H**_2_–CH=), 1.77 (tt, ^3^J = 6.7 Hz, ^3^J = 6.7 Hz, 2H, –OCH_2_–C**H**_2_–), 1.54 (tt, ^3^J = 7.7 Hz, ^3^J = 7.7 Hz, 2H, –CH_2_–); ^13^C NMR (126 MHz, Acetone-*d6*): δ = 169.62 (–(**C**=O)–CH_3_), 165.04 (Ar-(**C**=O)), 151.65 (C2), 139.33 (C5′), 134.63 (C4), 132.18 (C6), 126.78 (C5), 124.86 (C3), 124.75 (C1), 115.18 (C6′), 65.54 (C1′), 34.00 (C4′), 28.88 (C2′), 25.97 (C3′), 21.02 (–CH_3_).

### 4.5. General Procedure for the Synthesis of the Zeise’s Salt Derivatives ***1a***–***4a***

In an argon atmosphere, 1.0 equivalents of Zeise’s salt (0.30 mmol) was dissolved in 8 mL of degassed (three cycles freeze-pump-thaw), anhydrous ethanol under protection from light, and then stirred at room temperature. Then, 1.2 equivalents of the respective ligand (0.36 mmol), which was dissolved in approximately 2 mL of degassed and dry ethanol was added dropwise via syringe. After addition, the mixture was stirred at 48 °C for 3 h. The mixture was then allowed to cool to room temperature, upon which it was filtered and evaporated. Recrystallization from ethanol/diethyl ether afforded pure solid products. The crystals that were obtained in case of **1a** were suitable for X-ray analysis.

#### 4.5.1. Potassium {trichlorido[η^2^-(prop-2-en-1-yl)-2-acetoxybenzoate]platinate(II)} (Pt-Propene-ASA, **1a**)

Yield: 95% as yellow crystalline needles. m.p.: 94 °C (decomposition). HR-ESI-MS: calculated for C_12_H_12_Cl_3_O_4_Pt [**1a**–K]^−^: 520.9432. Found: *m*/*z* 520.9431. ^1^H NMR (400 MHz, Acetone-*d6*): δ = 8.10 (dd, ^3^J = 7.8 Hz, ^4^J = 1.7 Hz, 1H, ArH-6), 7.65 (ddd, ^3^J = 8.1 Hz, ^3^J = 7.4 Hz, ^4^J = 1.7 Hz, 1H, ArH-4), 7.39 (ddd, ^3^J = 7.6 Hz, ^3^J = 7.6 Hz, ^4^J = 1.2 Hz, 1H, ArH-5), 7.19 (dd, ^3^J = 8.1 Hz, ^4^J = 1.2 Hz, 1H, ArH-3), 5.00 (dddd, ^2^J_Pt-H_ = 62 Hz, ^3^J = 13.0 Hz, ^3^J = 7.7 Hz, ^3^J = 7.7 Hz, ^3^J = 5.5 Hz, 1H, –C**H**=CH_2_), 4.90 (dd, ^3^J_Pt-H_ = 32 Hz, ^2^J = 11.9 Hz, ^3^J = 5.5 Hz, 1H, –OC**H_α_**H_β_–), 4.50 (dd, ^3^J_Pt-H_ = 32 Hz, ^2^J = 11.9 Hz, ^3^J = 7.7 Hz, 1H, –OCH_α_**H_β_**–), 4.34 (dd, ^2^J_Pt-H_ = 73 Hz, ^3^J = 12.8 Hz, ^2^J = 1.4 Hz, 1H, =CH_2,_ *_trans_*), 4.31 (dd, ^2^J_Pt-H_ = 74 Hz, ^3^J = 7.9 Hz, ^2^J = 1.4 Hz, 1H, =CH_2,_ *_cis_*), 2.33 (s, 3H, –CH_3_); ^13^C NMR (101 MHz, Acetone-*d6*): δ = 169.84 (–(**C**=O)–CH_3_), 164.98 (Ar-(**C**=O)), 151.64 (C2), 134.67 (C4), 132.57 (C6), 126.79 (C5), 124.81 (C3), 124.68 (C1), 77.56 (C2′), 65.37 (C1′), 65.10 (C3′), 21.26 (–CH_3_).

#### 4.5.2. Potassium {trichlorido[η^2^-(but-3-en-1-yl)-2-acetoxybenzoate]platinate(II)} (Pt-Butene-ASA, **2a**)

Yield: 93% as yellow powder. m.p.: 118 °C (decomposition). HR-ESI-MS: calculated for C_13_H_14_Cl_3_O_4_Pt [**2a**–K]^−^: 535.9590. Found: *m*/*z* 535.9581. ^1^H NMR (400 MHz, Acetone-*d6*): δ = 8.08 (dd, ^3^J = 7.8 Hz, ^4^J = 1.8 Hz, 1H, ArH-6), 7.64 (ddd, ^3^J = 7.7 Hz, ^3^J = 7.7 Hz, ^4^J = 1.8 Hz, 1H, ArH-4), 7.39 (ddd, ^3^J = 7.6 Hz, ^3^J = 7.6 Hz, ^4^J = 1.2 Hz, 1H, ArH-5), 7.18 (dd, ^3^J = 8.1 Hz, ^4^J = 1.2 Hz, 1H, ArH-3), 5.16–4.87 (m, ^2^J_Pt-H_ = 62 Hz, 1H, –C**H**=CH_2_), 4.70–4.59 (m, 2H, –OCH_2_–), 4.38–4.11 (m, ^2^J_Pt-H_ = 60 Hz, 2H, =CH_2_), 2.57 (dddd, ^2^J = 14.1 Hz, ^3^J = 8.0 Hz, ^3^J = 6.0 Hz, ^3^J = 6.0 Hz, 1H, –C**H_α_**H_β_–), 2.30 (s, 3H, –CH_3_), 2.10–1.99 (m, 1H, –CH_α_**H_β_**–); ^13^C NMR (101 MHz, Acetone-*d6*): δ = 169.76 (–(**C**=O)–CH_3_), 165.11 (Ar-(**C**=O)), 151.68 (C2), 134.61 (C4), 132.43 (C6), 126.81 (C5), 124.81 (C3), 124.74 (C1), 84.18 (C3′), 65.96 (C4′), 64.86 (C1′), 33.36 (C2′), 21.15 (–CH_3_).

#### 4.5.3. Potassium {trichlorido[η^2^-(pent-4-en-1-yl)-2-acetoxybenzoate]platinate(II)} (Pt-Pentene-ASA, **3a**)

Yield: 57% as yellow powder. m.p.: 96 °C (decomposition). HR-ESI-MS: calculated for C_14_H_16_Cl_3_O_4_Pt [**3a**–K]^−^: 548.9734. Found: *m*/*z* 548.9771. ^1^H NMR (500 MHz, Acetone-*d6*): δ = 8.06 (dd, ^3^J = 7.9 Hz, ^4^J = 1.7 Hz, 1H, ArH-6), 7.64 (ddd, ^3^J = 8.1 Hz, ^3^J = 7.5 Hz, ^4^J = 1.8 Hz, 1H, ArH-4), 7.38 (ddd, ^3^J = 7.6 Hz, ^3^J = 7.6 Hz, ^4^J = 1.2 Hz, 1H, ArH-5), 7.18 (dd, ^3^J = 8.1 Hz, ^4^J = 1.2 Hz, 1H, ArH-3), 5.10–4.89 (m, ^2^J_Pt-H_ = 66 Hz, 1H, –C**H**=CH_2_), 4.35–4.33 (m, 2H, –OCH_2_–), 4.24–4.09 (m, ^2^J_Pt-H_ = 64 Hz, 2H, =CH_2_), 2.40–2.31 (m, 1H, 2′-H_α_), 2.30 (s, 3H, –CH_3_), 2.28–2.21 (m, 1H, 3′-H_α_), 2.15–2.07 (m, 1H, 2′-H_β_), 1.70 (dddd, ^2^J = 13.2 Hz, ^3^J = 9.3 Hz, ^3^J = 6.2 Hz, ^3^J = 6.2 Hz, 1H, 3′-H_β_); ^13^C NMR (126 MHz, Acetone-*d6*): δ = 169.75 (–(**C**=O)–CH_3_), 165.08 (Ar-(**C**=O)), 151.67 (C2), 134.56 (C4), 132.39 (C6), 126.77 (C5), 124.77 (C3), 88.91 (C4′), 65.43 (C1′), 65.05 (C5′), 30.60 (C3′), 29.04 (C2′), 21.13 (–CH_3_); C1 not observed.

#### 4.5.4. Potassium {trichlorido[η^2^-(hex-5-en-1-yl)-2-acetoxybenzoate]platinate(II)} (Pt-Hexene-ASA, **4a**)

Yield: 19% as yellow powder. m.p.: 100 °C (decomposition). HR-ESI-MS: calculated for C_15_H_18_Cl_3_O_4_Pt [**4a**–K]^−^: 562.9891. Found: *m*/*z* 562.9928. ^1^H NMR (500 MHz, Acetone-*d6*): δ = 8.06 (dd, ^3^J = 7.9 Hz, ^4^J = 1.7 Hz, 1H, ArH-6), 7.63 (ddd, ^3^J = 7.7 Hz, ^3^J = 7.7 Hz, ^4^J = 1.7 Hz, 1H, ArH-4), 7.40 (ddd, ^3^J = 7.6 Hz, ^3^J = 7.6 Hz, ^4^J = 1.2 Hz, 1H, ArH-5), 7.18 (dd, ^3^J = 8.2 Hz, ^4^J = 1.2 Hz, 1H, ArH-3), 5.10–4.84 (m, ^2^J_Pt–H_ = 60 Hz, 1H, –C**H**=CH_2_), 4.37–4.25 (m, 2H, –OCH_2_–), 4.25–4.04 (m, ^2^J_Pt-H_ = 59 Hz, 2H, =CH_2_), 2.29 (s, 3H, –CH_3_), 2.27–2.19 (m, 1H, 4′-H_α_), 2.03–1.97 (m, 1H, 2′-H_α_), 1.94–1.87 (m, 1H, 3′-H_α_), 1.88–1.81 (m, 1H, 3′-H_β_), 1.81–1.71 (m, 1H, 2′-H_β_), 1.65 (ddt, ^2^J = 13.1 Hz, ^3^J = 9.0 Hz, ^3^J = 6.5 Hz, 1H, 4′-H_β_); ^13^C NMR (126 MHz, Acetone-*d6*): δ = 169.71 (–(**C**=O)–CH_3_), 165.13 (Ar-(**C**=O)), 151.62 (C2), 134.51 (C4), 132.44 (C6), 126.88 (C5), 124.82 (C1), 124.75 (C3), 89.60 (C5′), 65.69 (C1′), 64.84 (C6′), 33.80 (C4′), 29.15 (C2′), 26.36 (C3′), 21.12 (–CH_3_).

### 4.6. General Methods for the Cell Culture

The HT-29 colon carcinoma and the MCF-7 breast cancer cell lines were obtained from the cell line service (CLS, Eppelheim, Germany). The HT-29 and the MCF-7 cells were cultivated as a monolayer in Dulbecco’s Modified Eagle Medium (DMEM) containing glucose (4.5 g/L) and lacking phenol red (GE Healthcare, Little Chalfont, UK), supplemented with fetal calf serum (FCS; 10%; Biochrom) and l-glutamine (584 mg/L; GE Healthcare). The cells were cultured in a humidified atmosphere (5% CO_2_/95% air) at 37 °C and were passaged regularly twice a week. The cell lines were characterized by typing short tandem repeats and routinely monitored for mycoplasma infection.

#### 4.6.1. COX-1/2 Isoenzyme Inhibition

The inhibition of both isolated isoenzymes, ovine COX-1 and ovine COX-2, respectively, were investigated at a concentration of 10 µM of the respective substances while employing an enzyme immunoassay (COX Inhibitor Screening Assay, Cayman Chemicals, Ann Arbor, MI, USA). The conduction of the assay was performed following the manufacturer’s protocol. Thereby, the isoenzymes were incubated with the particular compounds for exactly 10 min. The values express the mean of duplicate determinations and they represent the mean ± standard deviation of ≥2 independent experiments. The untreated control was set to 0% inhibition.

#### 4.6.2. Antiproliferative Effects

The effects of the newly synthesized substances on the cell biomass were determined in vitro while employing a crystal violet assay. The testing was performed following a modified protocol, as previously published [26]. Exponentially growing HT-29 and MCF-7 cells were seeded in 96-well tissue culture plates with a density of 3 × 10^3^ and 2 × 10^3^ cells, respectively, in 100 µL of completed DMEM per well in quadruples. The plates were kept in a humidified atmosphere (5% CO_2_/95% air) at 37 °C for 24 h prior to the addition of completed medium containing the vehicle DMF, the positive control Cisplatin, and the compounds to be tested, respectively. After 72 h of incubation, the medium was removed, the cells were washed with PBS and fixed using a solution of glutaraldehyde in PBS (1%, *v*/*v*). The cell biomass was examined by staining the chromatin of adherent cells with crystal violet, extraction of the stain with ethanol (70%, *v*/*v*) and following measurement of absorbance (λ = 590 nm). The cell viability is given as percentage of cell viability of the vehicle treated control, which was set to 100%. The IC_50_ values represent the mean ± standard error of mean of ≥2 independent experiments and they were calculated with Prism 7.0 (GraphPad, San Diego, CA, USA) using nonlinear regression and decadal logarithm of the inhibitor versus variable slope equation. The top constraint was set to 100%.

## Figures and Tables

**Table 1:** Selected bond lengths and bond angles in the crystal of **1a**.

Structural Element	Bond Length/Å	Bond Angle/°
Pt(1)–C(1)	2.114(3)	
Pt(1)–C(2)	2.155(3)	
Pt(1)–Cl(3) (*cis*)	2.3030(7)	
Pt(1)–Cl(2) (*cis*)	2.3033(7)	
Pt(1)–Cl(1) (*trans*)	2.3244(7)	
C(1)–C(2)	1.405(4)	
C(2)–C(3)	1.484(4)	
C(1)–C(2)–C(3)		119.0(3)
C(1)–C(2)–H(2)		120.0(2)
C(3)–C(2)–H(2)		115.0(2)
Pt(1)–C(2)–C(1)		69.2(2)
Pt(1)–C(2)–C(3)		119.5(2)
Pt(1)–C(2)–H(2)		104.6(2)

**Table 2:** Selected ^1^H and ^13^C chemical shifts of the vinyl group of the ligands **1**–**4** and the corresponding complexes **1a**–**4a** ^1^.

Compound	^1^H NMR				^13^C NMR			
	–CH=CH_2_	Δδ	–CH=CH_2_	Δδ	–CH=CH_2_	Δδ	–CH=CH_2_	Δδ
**1**	6.06	1.06	5.42/5.28	1.08/0.97	133.43	55.87	118.65	53.55
**1a**	5.00		4.34/4.31		77.56		65.10	
**2**	5.90	0.88	5.18/5.08	0.93/0.83	135.26	51.08	117.59	51.63
**2a**	5.02		4.25		84.18		65.96	
**3**	5.88	0.88	5.08/4.99	0.91/0.82	138.59	49.68	115.59	50.54
**3a**	5.00		4.17		88.91		65.05	
**4**	5.84	0.87	5.04/4.95	0.89/0.80	139.33	49.73	115.18	50.34
**4a**	4.97		4.15		89.60		64.84	

^1^ Spectra measured in Acetone-*d6*. Chemical shifts (δ) are given in ppm.

**Table 3:** Inhibition of the cell growth in colon carcinoma (HT-29) and breast cancer (MCF-7) cell lines determined in a crystal violet cell biomass assay.

Compound	IC_50_/µM ^1^	
	HT-29	MCF-7
**ASA**	≥50	≥50
**Zeise’s salt**	≥50	≥50
**1a**	49.7 ± 1.8	≥50
**2a**	31.4 ± 0.4	30.1 ± 1.5
**3a**	44.2 ± 3.4	37.4 ± 2.5
**4a**	41.4 ± 0.3	43.7 ± 5.7
**Cisplatin**	2.6 ± 0.1	3.7 ± 0.3

^1^ IC_50_ values represent the concentration where 50% of the compounds maximum antiproliferative activity compared to vehicle treated control was achieved. Results are given as mean ± standard error of mean of ≥2 independent experiments.

## Supplementary Materials

Supplementary materials can be found at http://www.mdpi.com/1422-0067/19/6/1612/s1.

## Abbreviations

ASA	acetylsalicylic acid
BGE	background electrolyte
CCDC	Cambridge Crystallographic Data Centre
CE	capillary electrophoresis
COX	cyclooxygenase
DAD	diode array detector
DCC	*N,N*′-dicyclohexylcarbodiimide
DMAP	4-dimethylaminopyridine
DMF	dimethylformamide
DMSO	dimethyl sulfoxide
HR-ESI-MS	high resolution electrospray ionization mass spectrometry
m.p.	melting point
NMR	nuclear magnetic resonance
NSAID	nonsteroidal anti-inflammatory drug
PBS	phosphate-buffered saline
SA	salicylic acid
TLC	thin layer chromatography
TMS	tetramethylsilane
τ_1/2_	half-live
