# Supplementary material for: Synthesis and Biological Evaluation of Zeise’s Salt Derivatives with Acetylsalicylic Acid Substructure
Source: Int J Mol Sci. 2018 May 30;19(6):1612. doi: 10.3390/ijms19061612 (PMC6032411; doi:10.3390/ijms19061612)
Supplement: Supplementary file 1 [file ijms-19-01612-s001.pdf]

Supplementary Materials

Synthesis and Biological Evaluation of Zeise's Salt Derivatives with Acetylsalicylic Acid Substructure

Alexander Weninger [1], Daniel Baecker [1], Victoria Obermoser [1], Dorothea Egger [1], Klaus Wurst [2] and Ronald Gust [1,*]

1 Department of Pharmaceutical Chemistry, Institute of Pharmacy, Center for Molecular Biosciences Innsbruck, University of Innsbruck, CCB – Centrum for Chemistry and Biomedicine, Innrain 80-82, 6020 Innsbruck, Austria; alexander.weninger@uibk.ac.at (A.W.); daniel.baecker@uibk.ac.at (D.B.); victoria.obermoser@gmx.at (V.O.); dorothea.egger@gmx.at (D.E.); ronald.gust@uibk.ac.at (R.G.)
2 Institute of General, Inorganic and Theoretical Chemistry, University of Innsbruck, CCB – Centrum for Chemistry and Biomedicine, Innrain 80-82, 6020 Innsbruck, Austria; klaus.wurst@uibk.ac.at (K.W.)
* Correspondence: ronald.gust@uibk.ac.at; Tel.: +43-512-507-58200

1. HR-ESI-MS

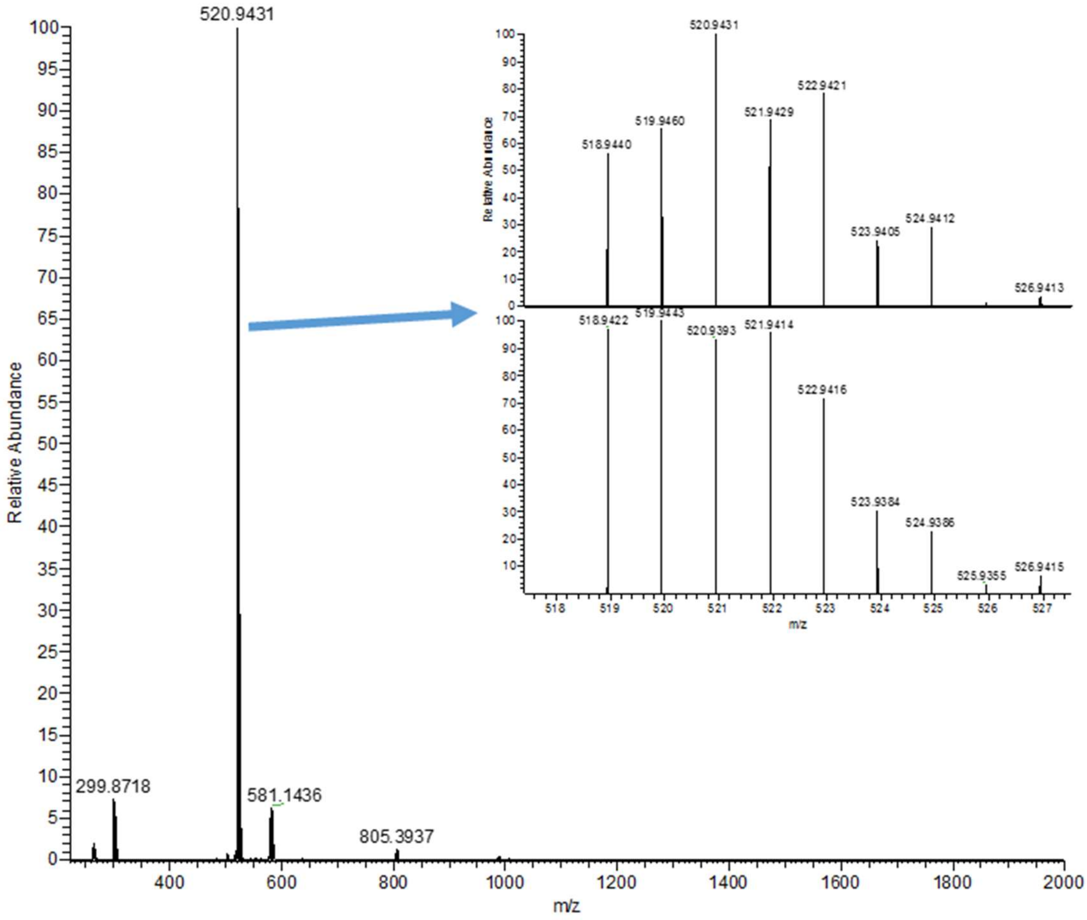

Figure S1. HR-ESI-MS spectrum of Pt-Propene-ASA (**1a**), recorded in negative mode; The inset shows the peak of [**1a**-K]⁻ with the characteristic line pattern of the platinum isotopes (top) and the calculated peak (bottom)

2. 1D and 2D NMR spectra

2.1 Propene-ASA (1)

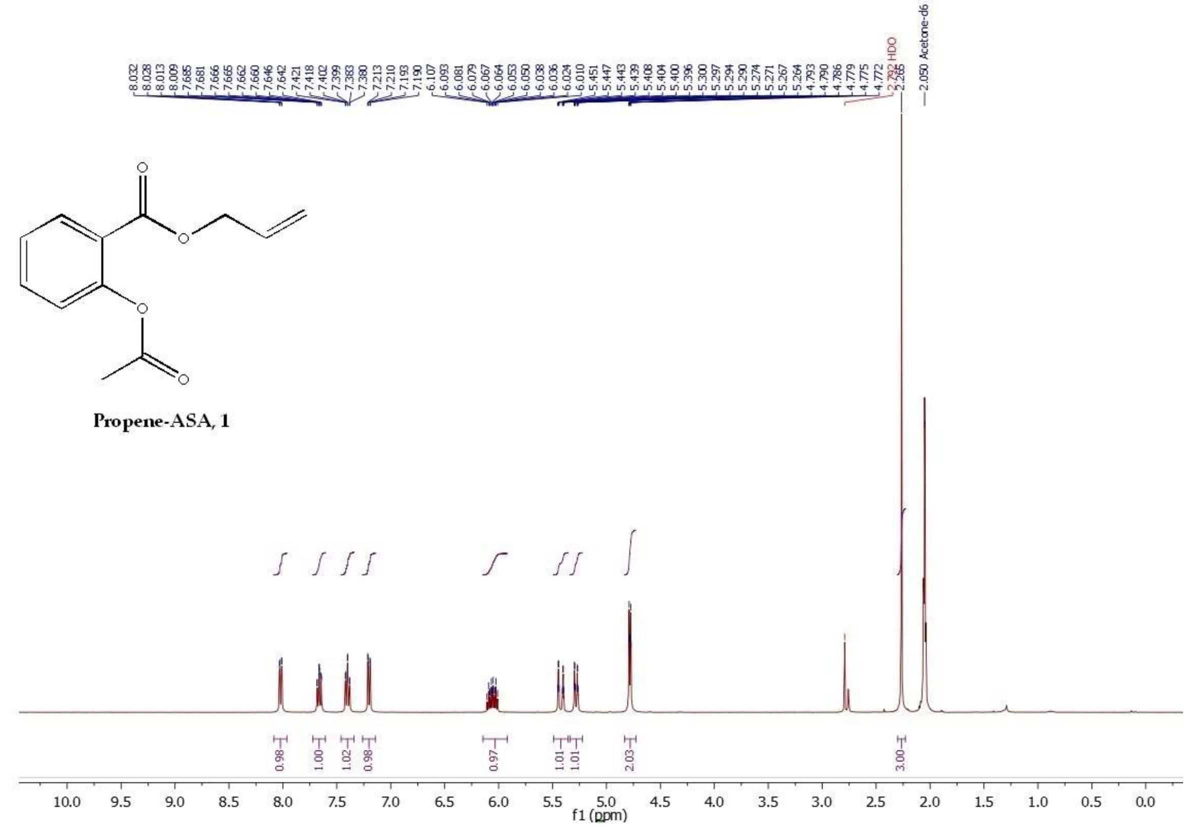

Figure S2. ^{1}H NMR of Propene-ASA (**1**) in Acetone-*d6*

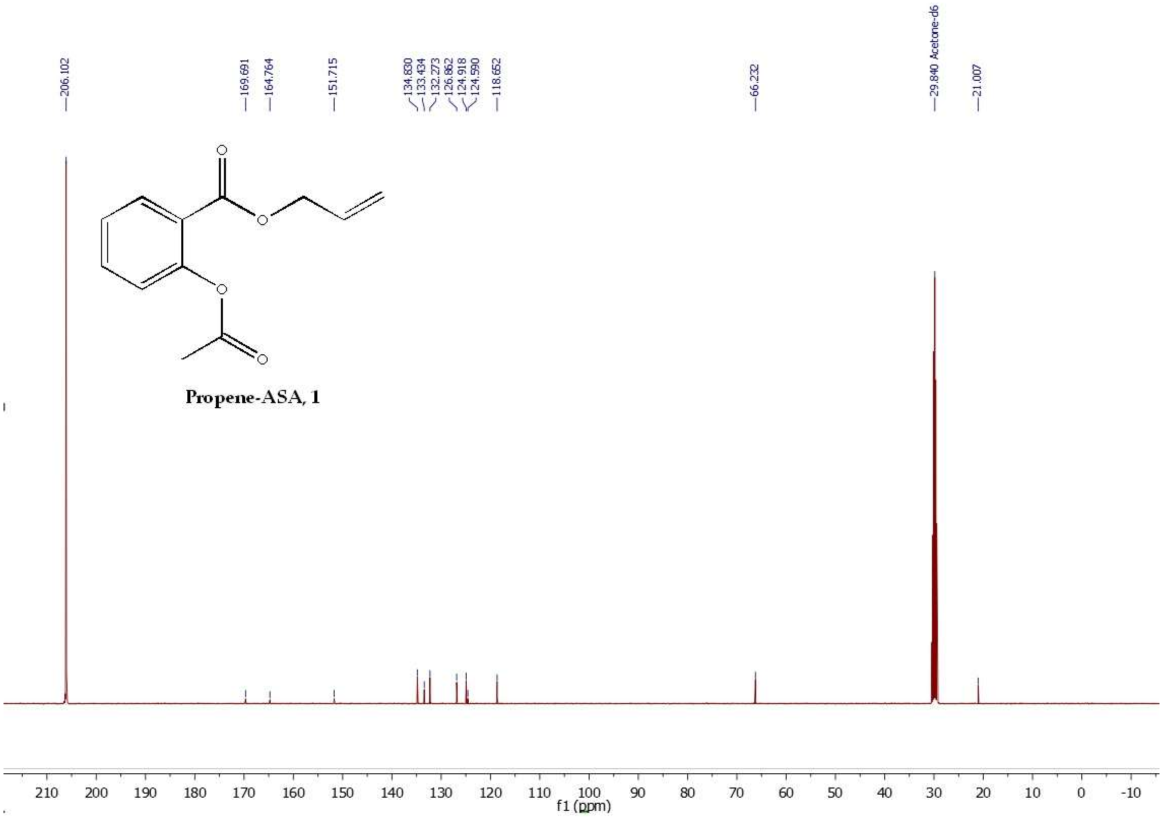

Figure S3. ^{13}C NMR of Propene-ASA (**1**) in Acetone-*d6*

2.2 Pt-Propene-ASA (1a)

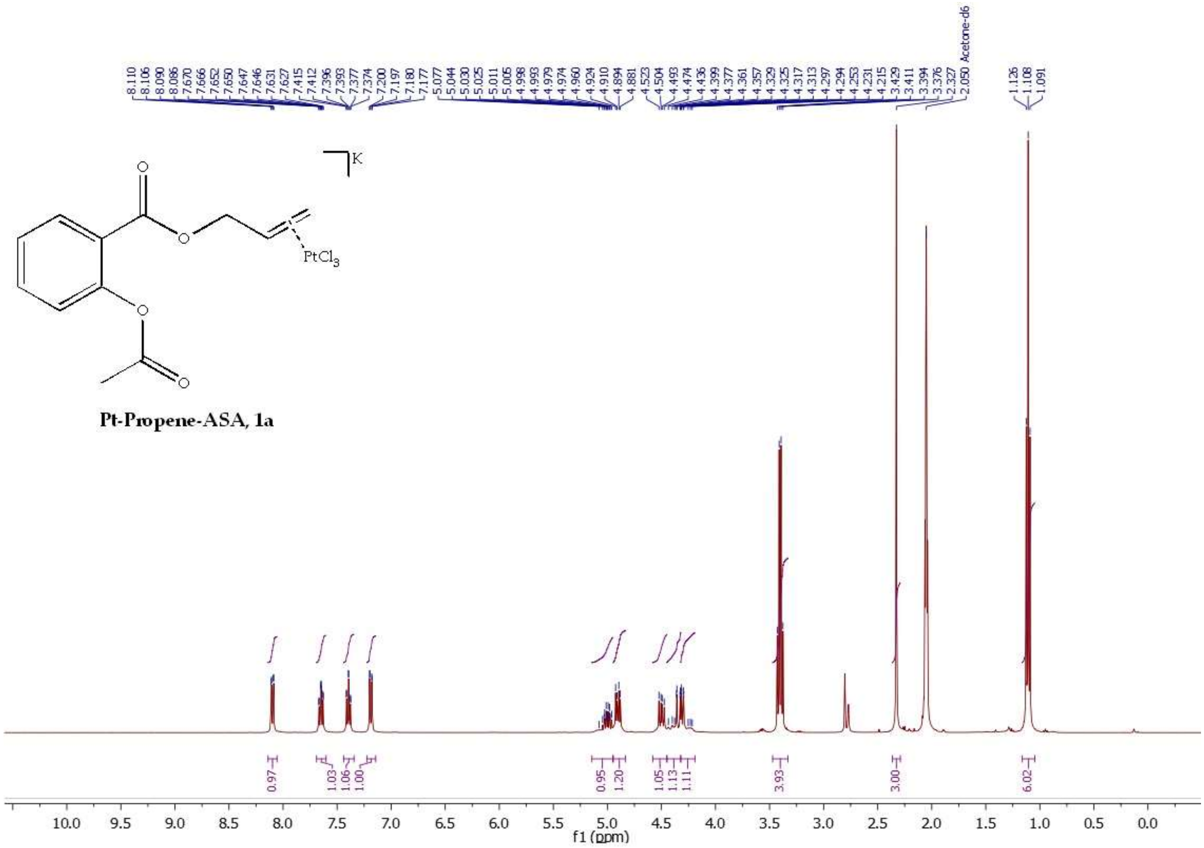

Figure S4. ^{1}H NMR of Pt-Propene-ASA (**1a**) in Acetone-*d6*

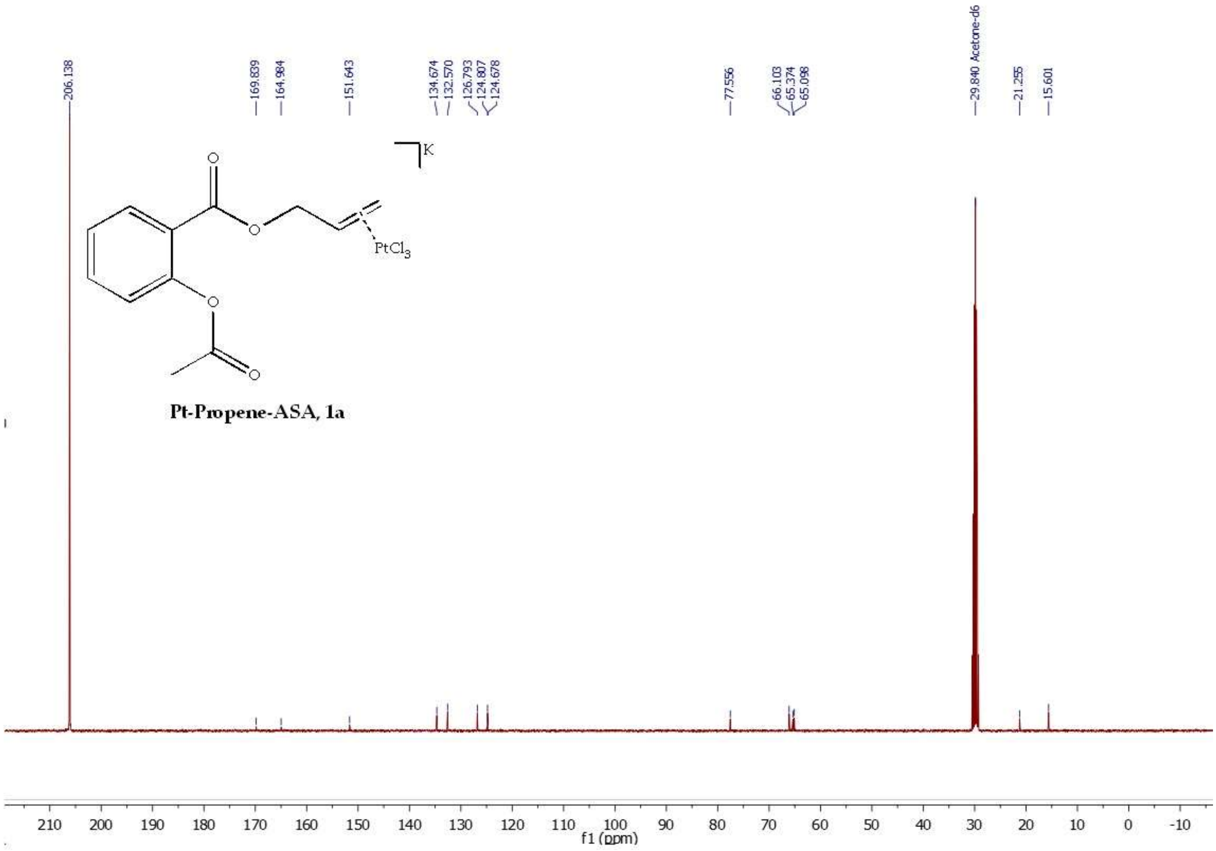

Figure S5. ^{13}C NMR of Pt-Propene-ASA (**1a**) in Acetone-*d6*

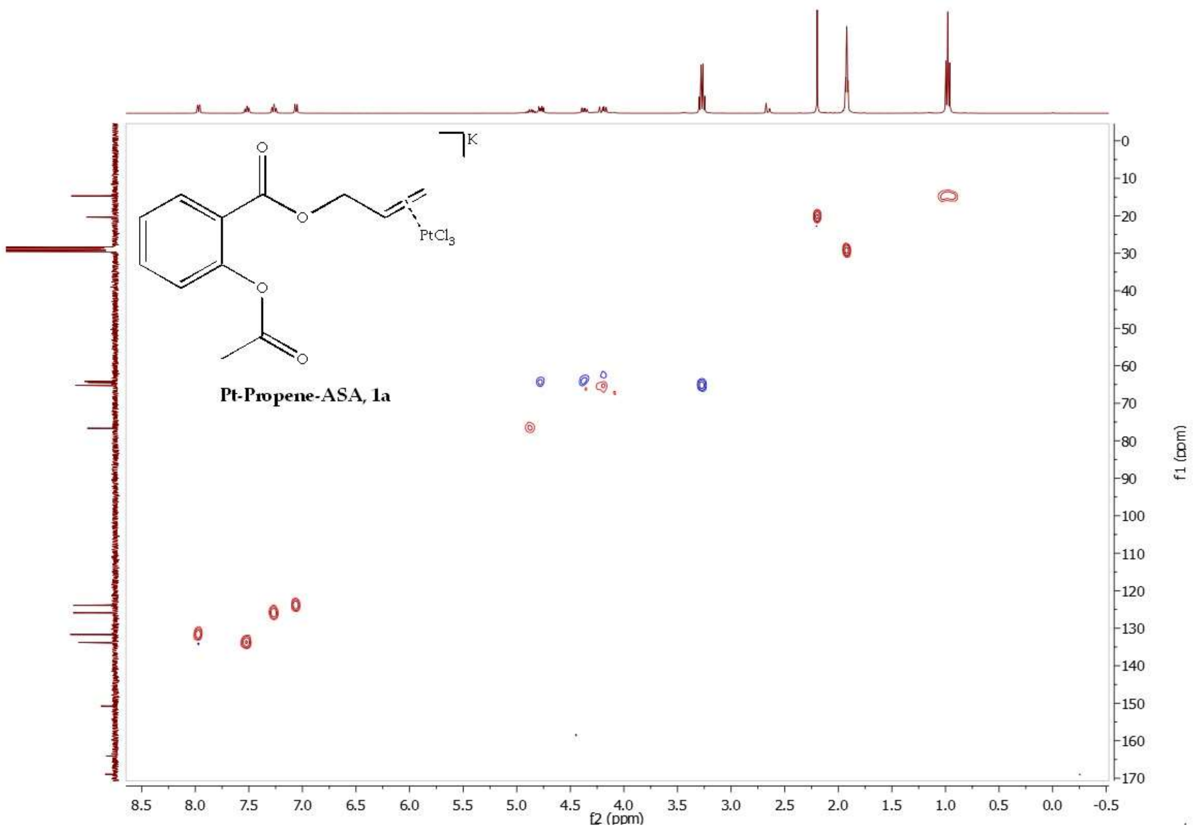

Figure S6. [^{1}H,^{13}C]-HSQC of Pt-Propene-ASA (**1a**) in Acetone-*d6*

2.3 Butene-ASA (2)

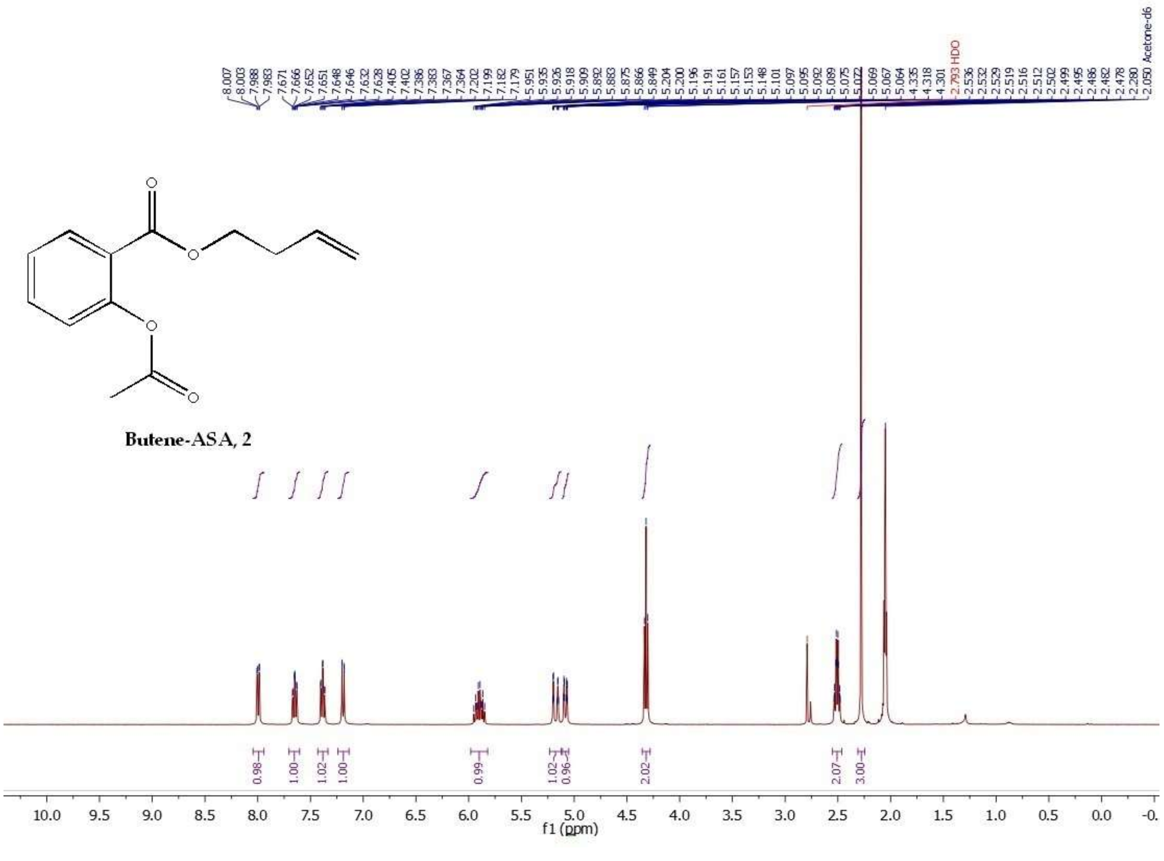

Figure S7. ^{1}H NMR of Butene-ASA (**2**) in Acetone-*d6*

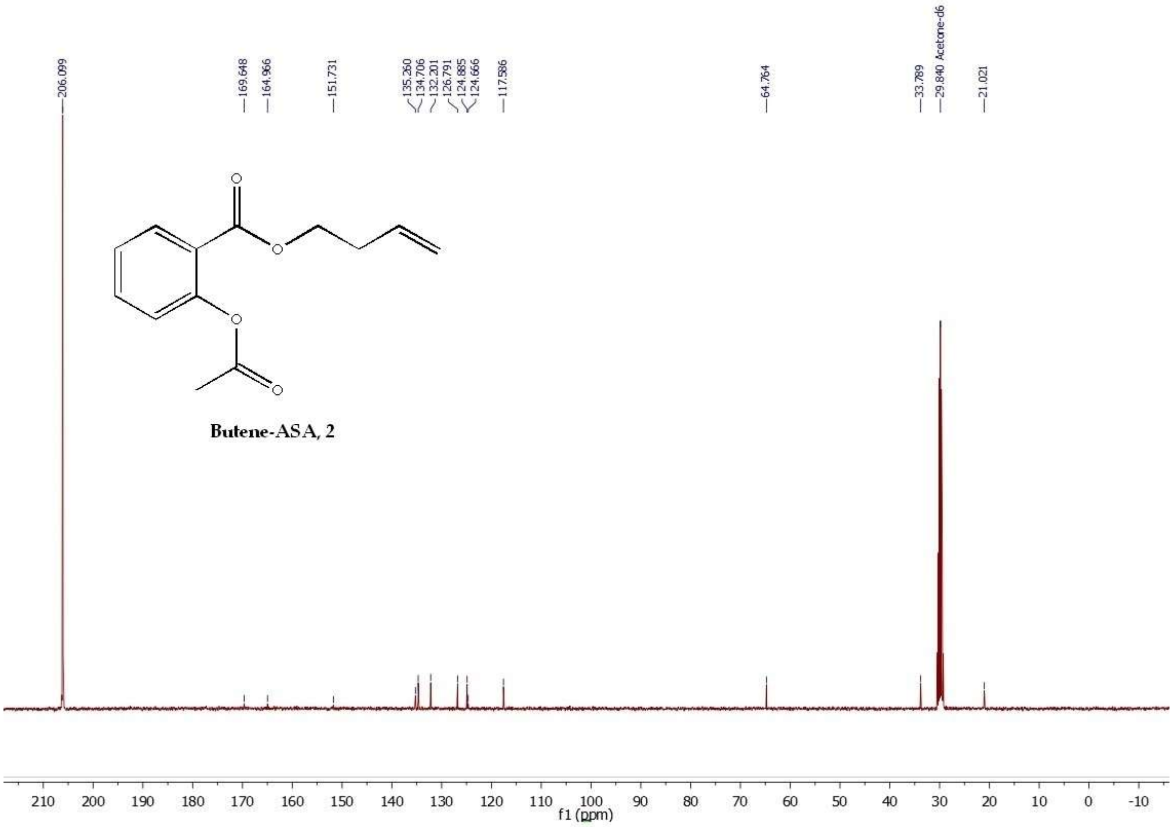

Figure S8. ^{13}C NMR of Butene-ASA (**2**) in Acetone-*d6*

2.4 Pt-Butene-ASA (2a)

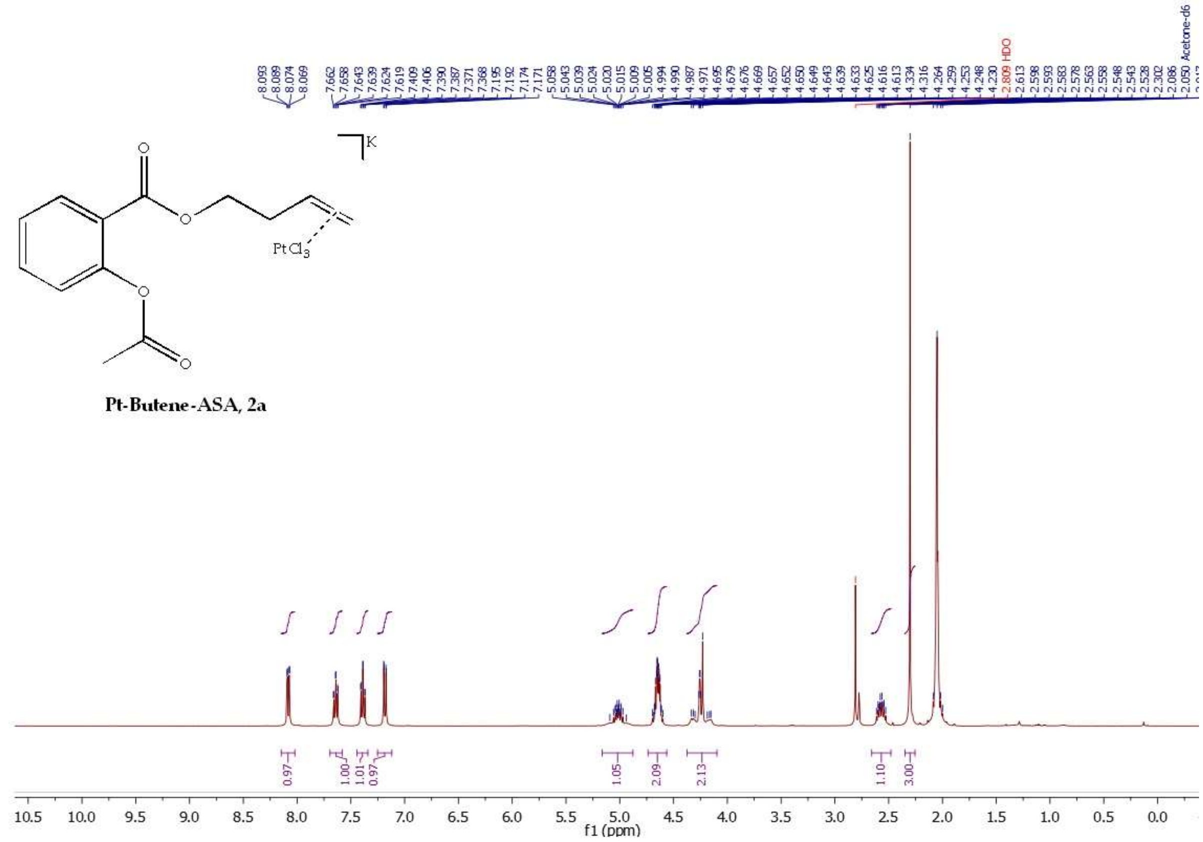

Figure S9. ^{1}H NMR of Pt-Butene-ASA (**2a**) in Acetone-*d6*

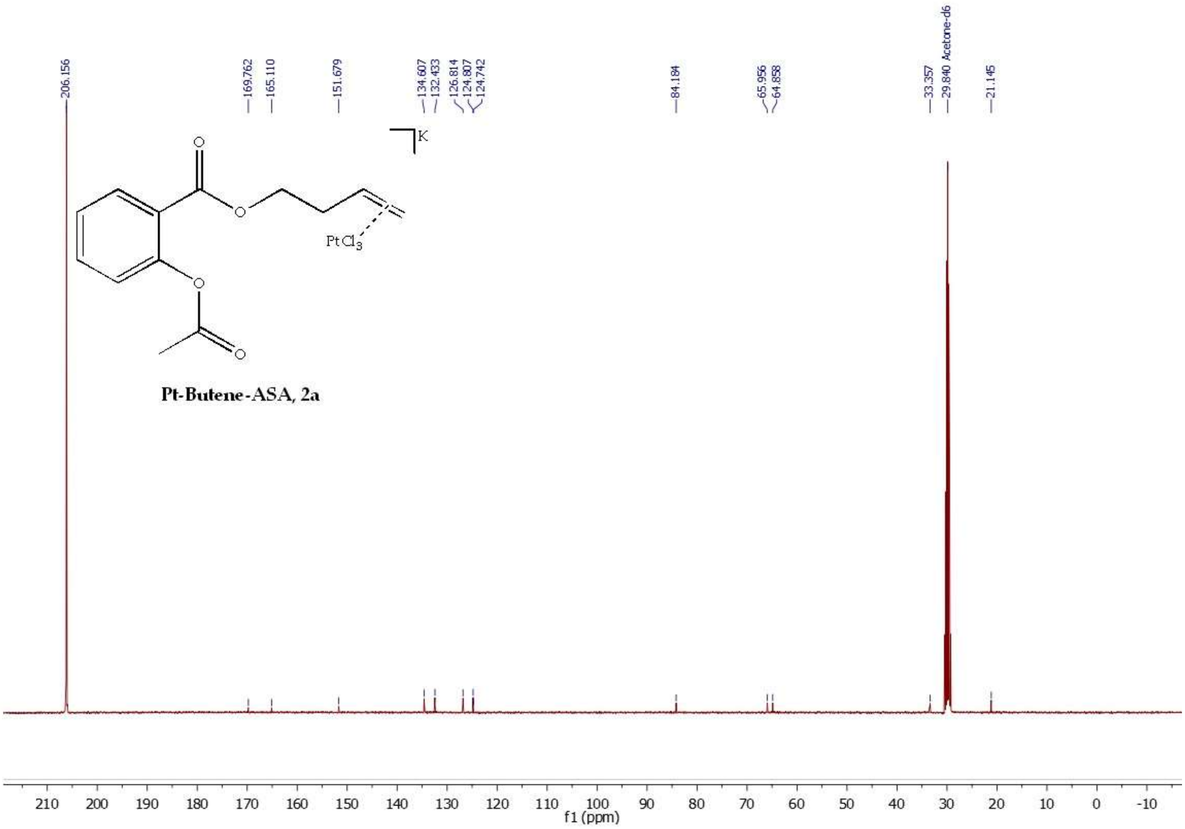

Figure S10. ^{13}C NMR of Pt-Butene-ASA (**2a**) in Acetone-*d6*

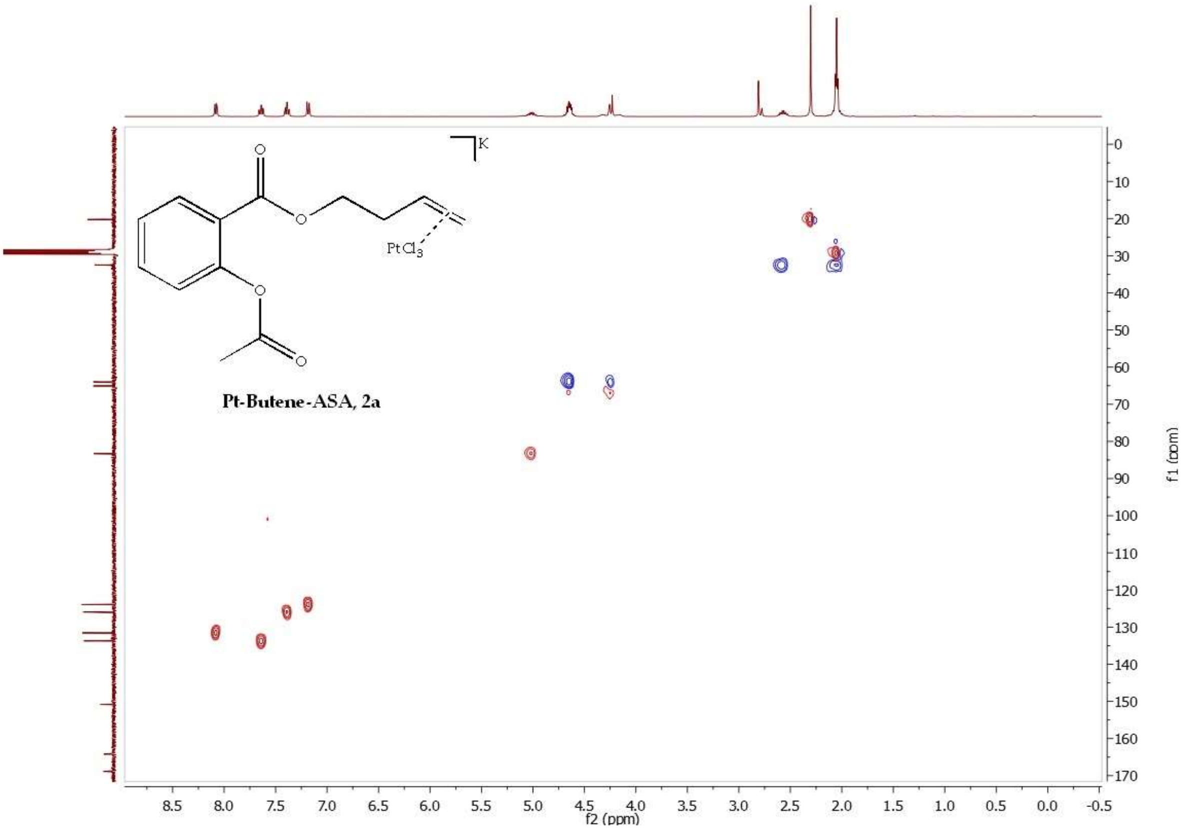

Figure S11. [^{1}H,^{13}C]-HSQC of Pt-Butene-ASA (**2a**) in Acetone-*d6*

2.5 Pentene-ASA (3)

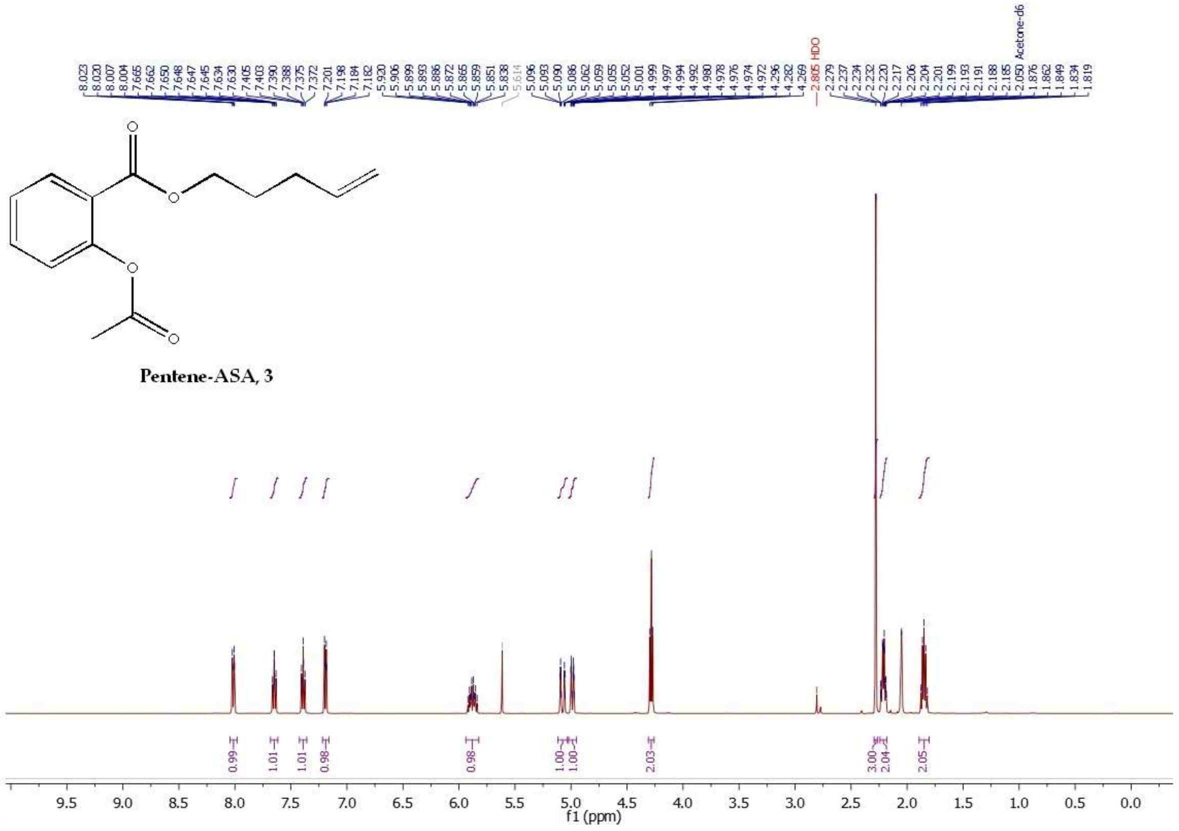

Figure S12. ^{1}H NMR of Pentene-ASA (**3**) in Acetone-*d6*

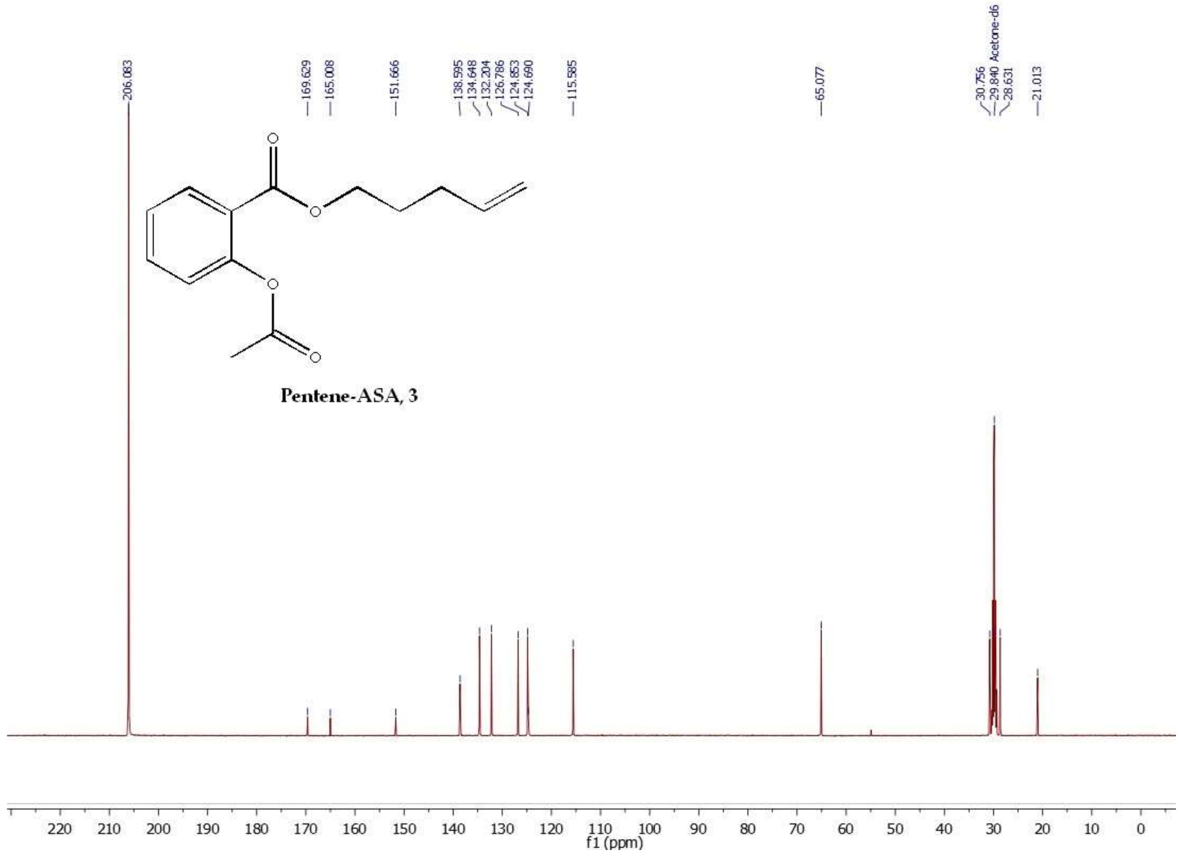

Figure S13. ^{13}C NMR of Pentene-ASA (**3**) in Acetone-*d6*

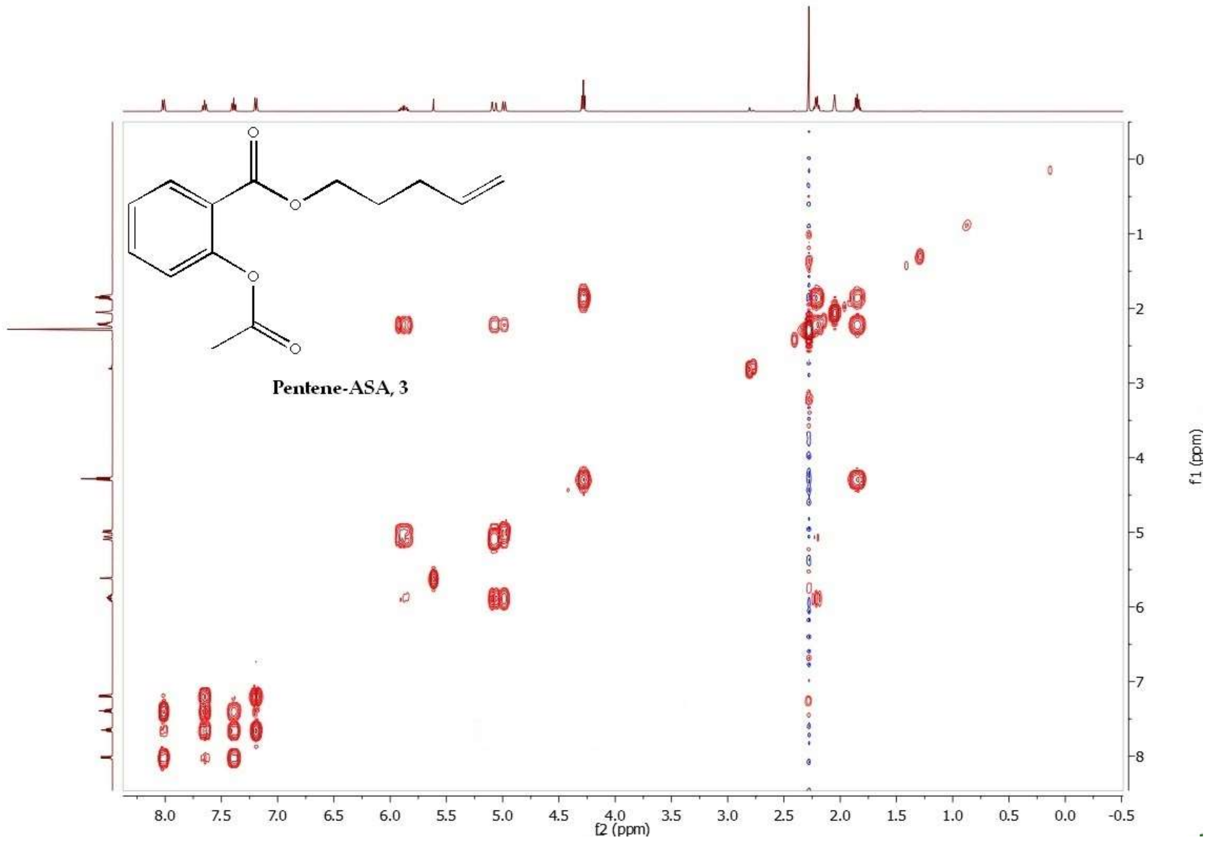

Figure S14. [^{1}H,^{1}H]-COSY of Pentene-ASA (**3**) in Acetone-*d6*

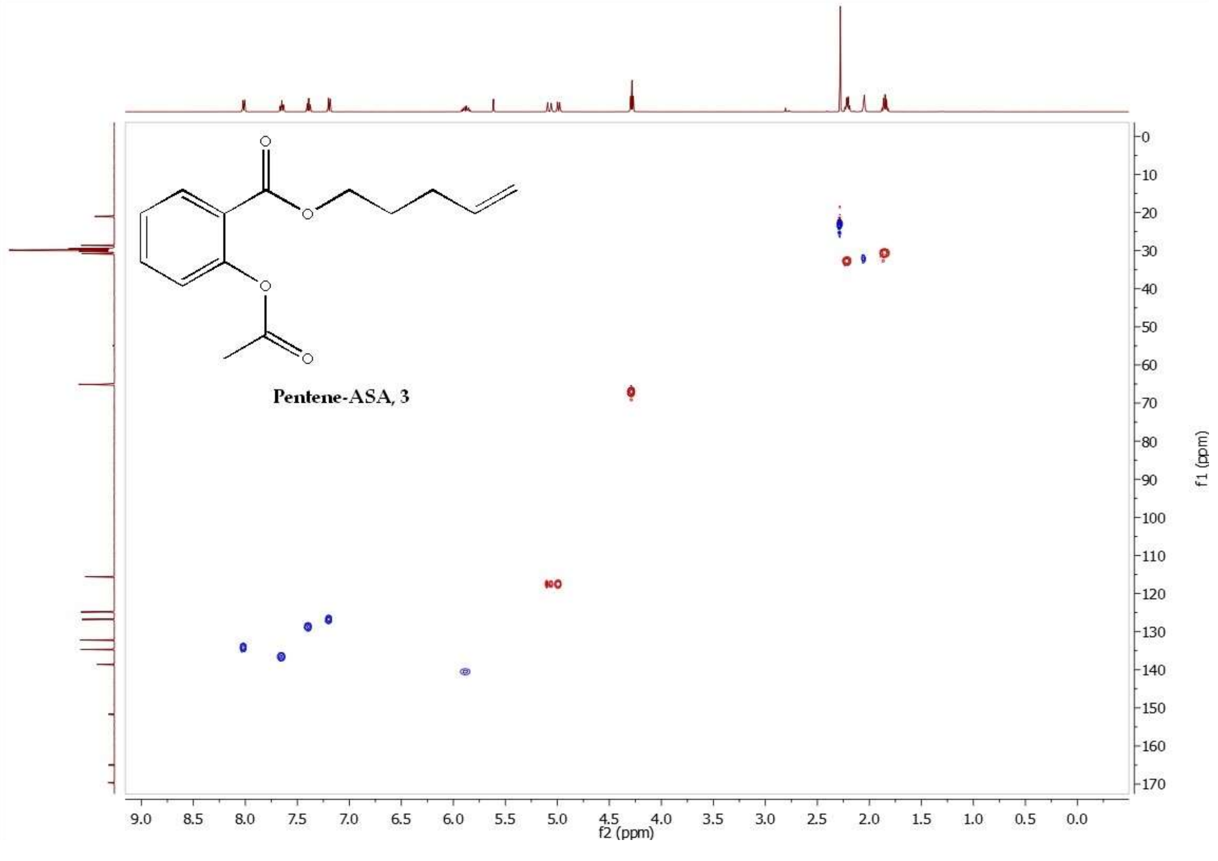

Figure S15. [^{1}H,^{13}C]-HSQC of Pentene-ASA (**3**) in Acetone-*d6*

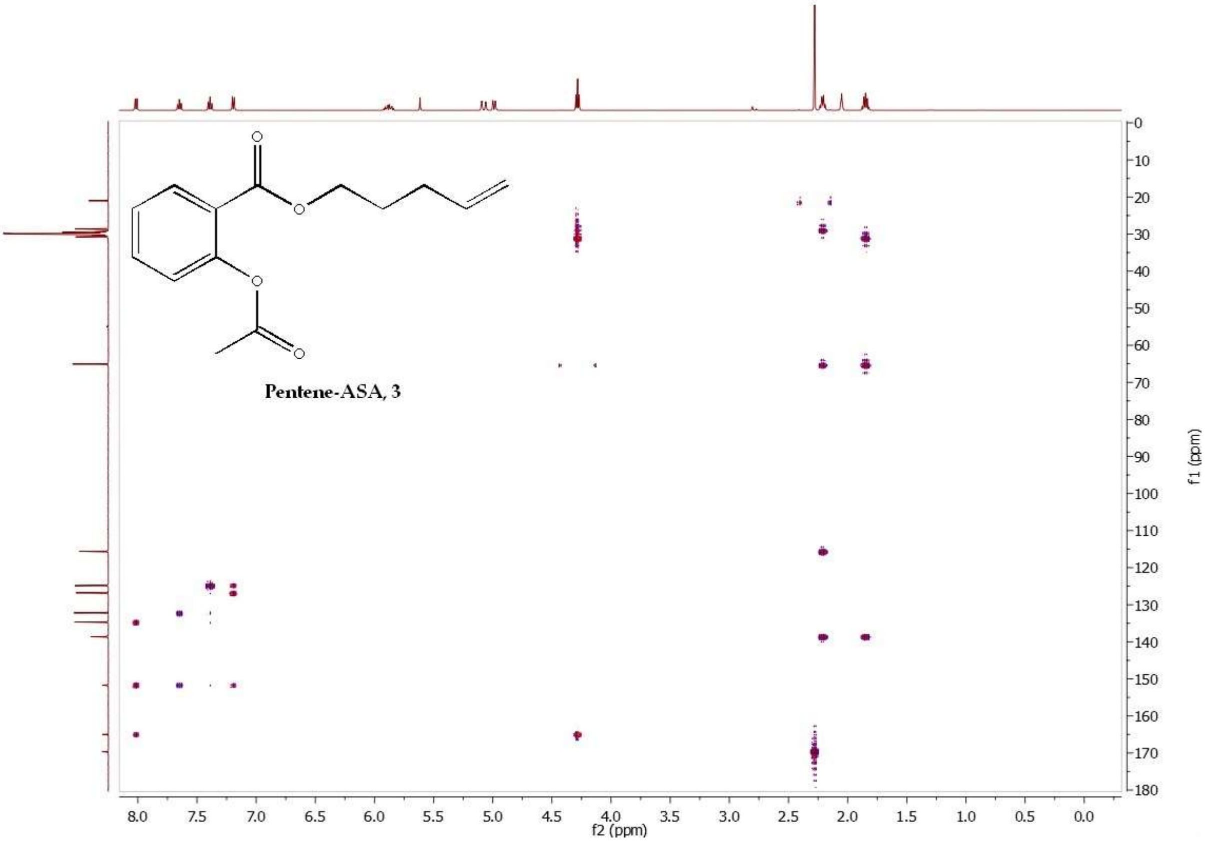

Figure S16. [^{1}H,^{13}C]-HMBC of Pentene-ASA (**3**) in Acetone-*d6*

2.6 Pt-Pentene-ASA (3a)

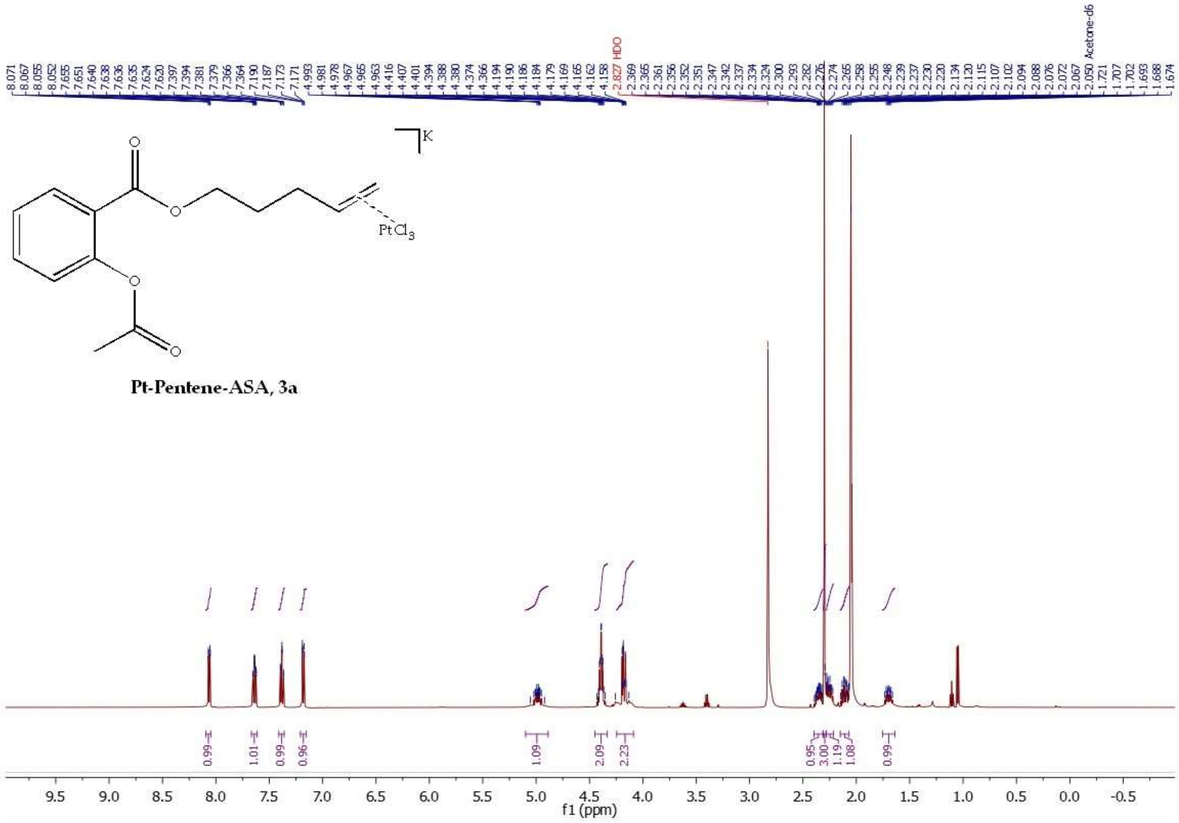

Figure S17. ^{1}H NMR of Pt-Pentene-ASA (**3a**) in Acetone-*d6*

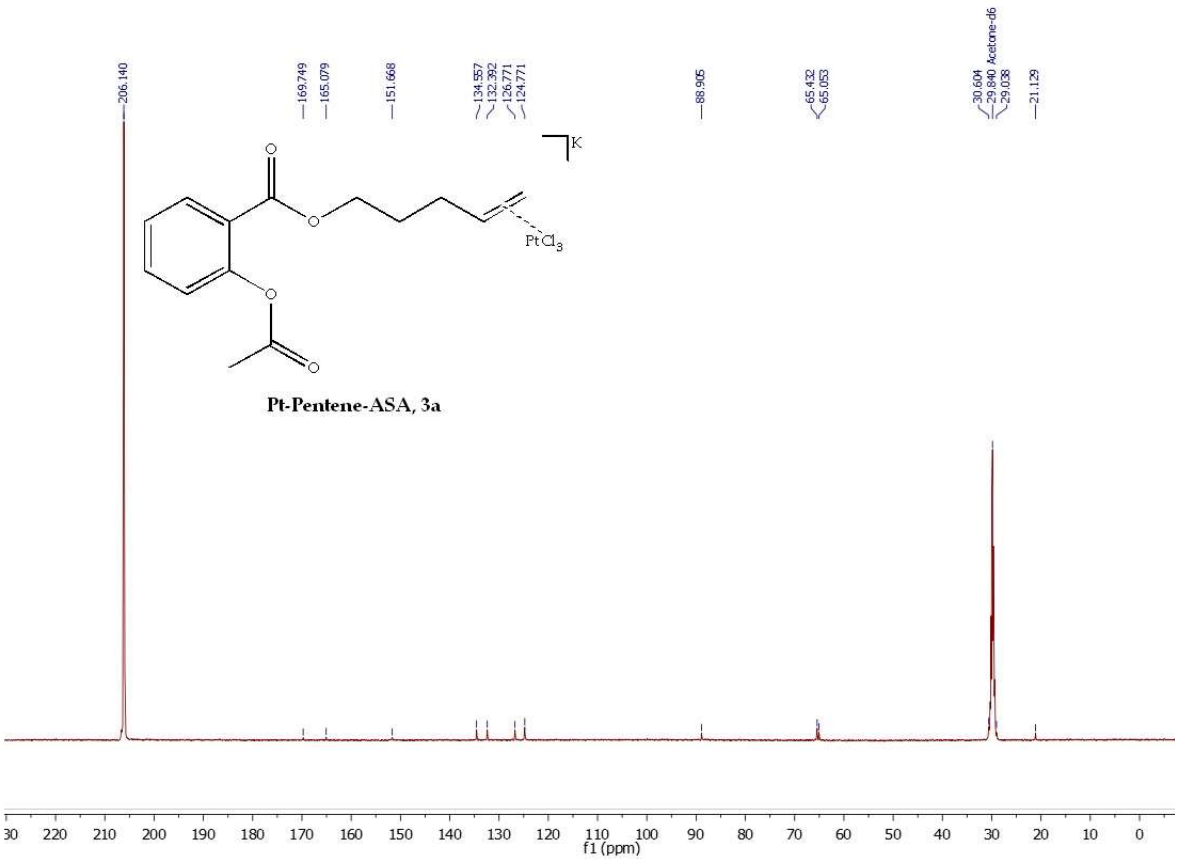

Figure S18. ^{13}C NMR of Pt-Pentene-ASA (**3a**) in Acetone-*d6*

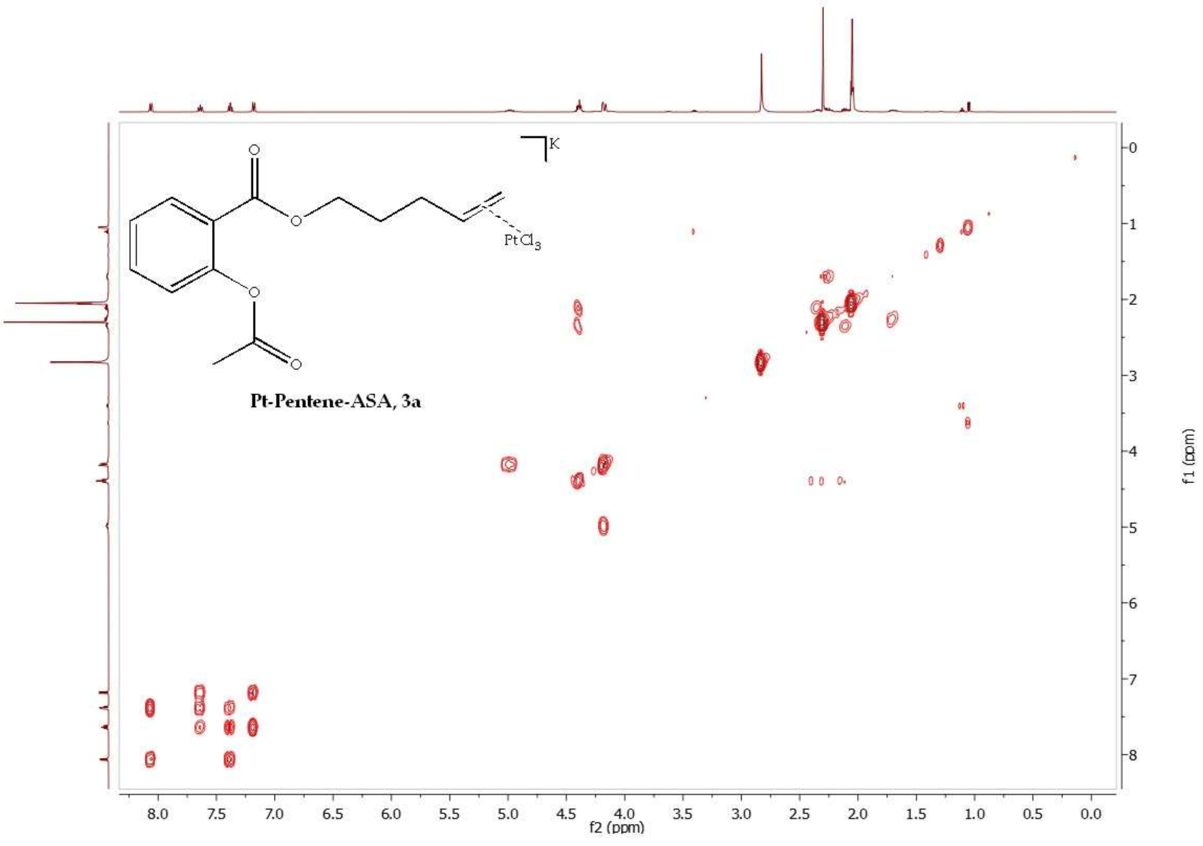

Figure S19. [^{1}H,^{1}H]-COSY of Pt-Pentene-ASA (**3a**) in Acetone-*d6*

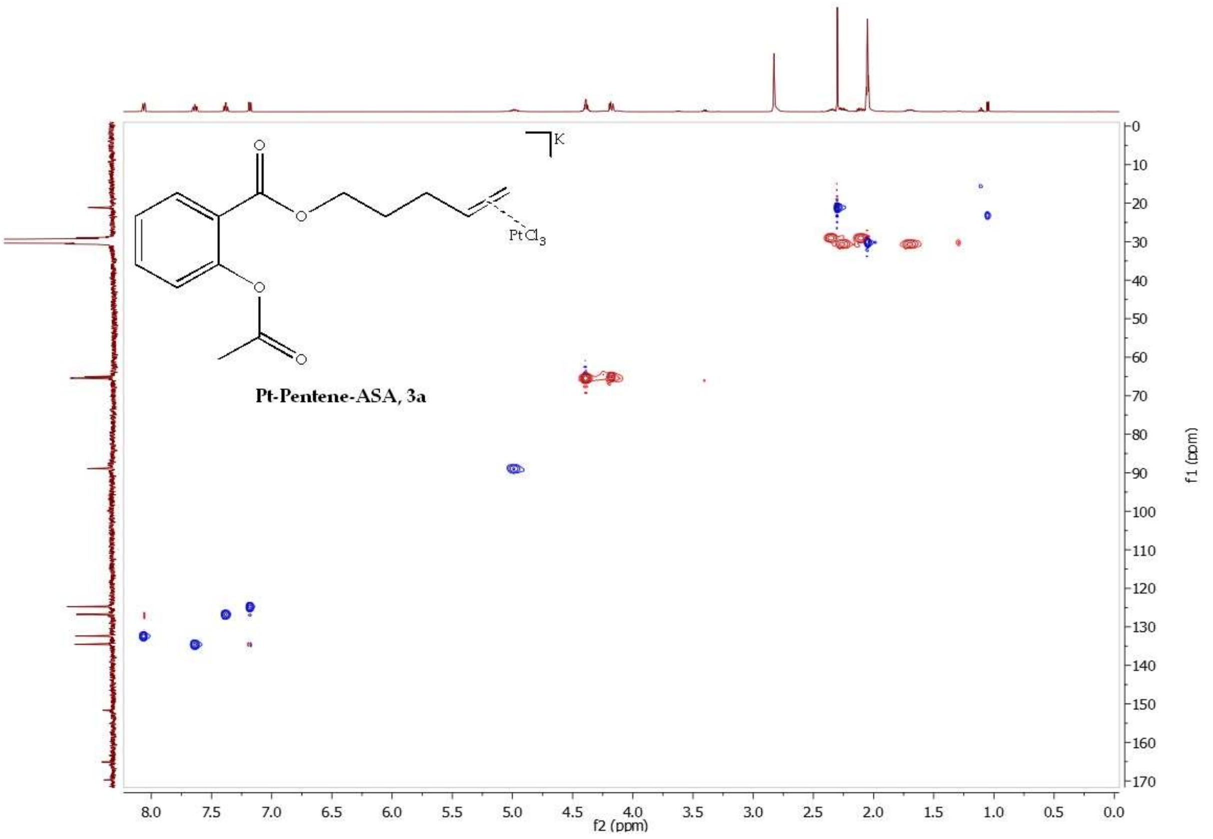

Figure S20. [^{1}H,^{13}C]-HSQC of Pt-Pentene-ASA (**3a**) in Acetone-*d6*

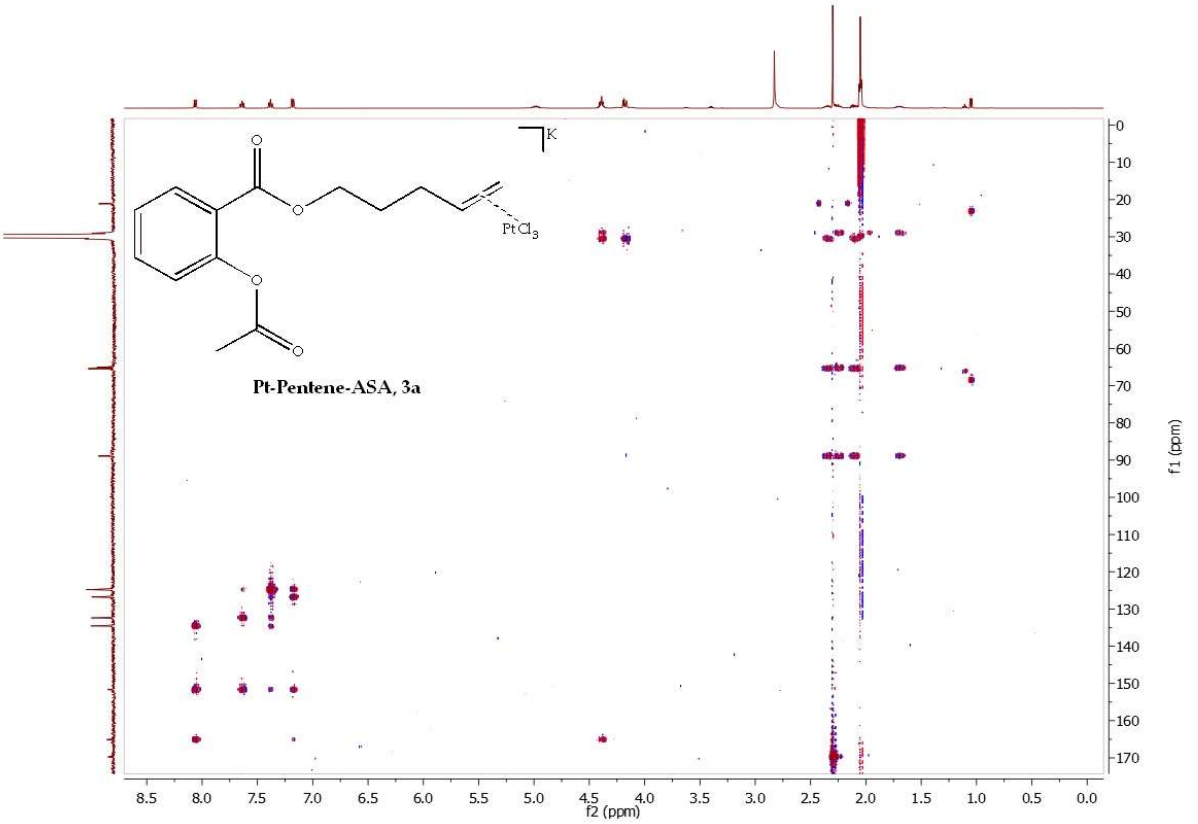

Figure S21. [^{1}H,^{13}C]-HMBC of Pt-Pentene-ASA (**3a**) in Acetone-*d6*

2.7 Hexene-ASA (4)

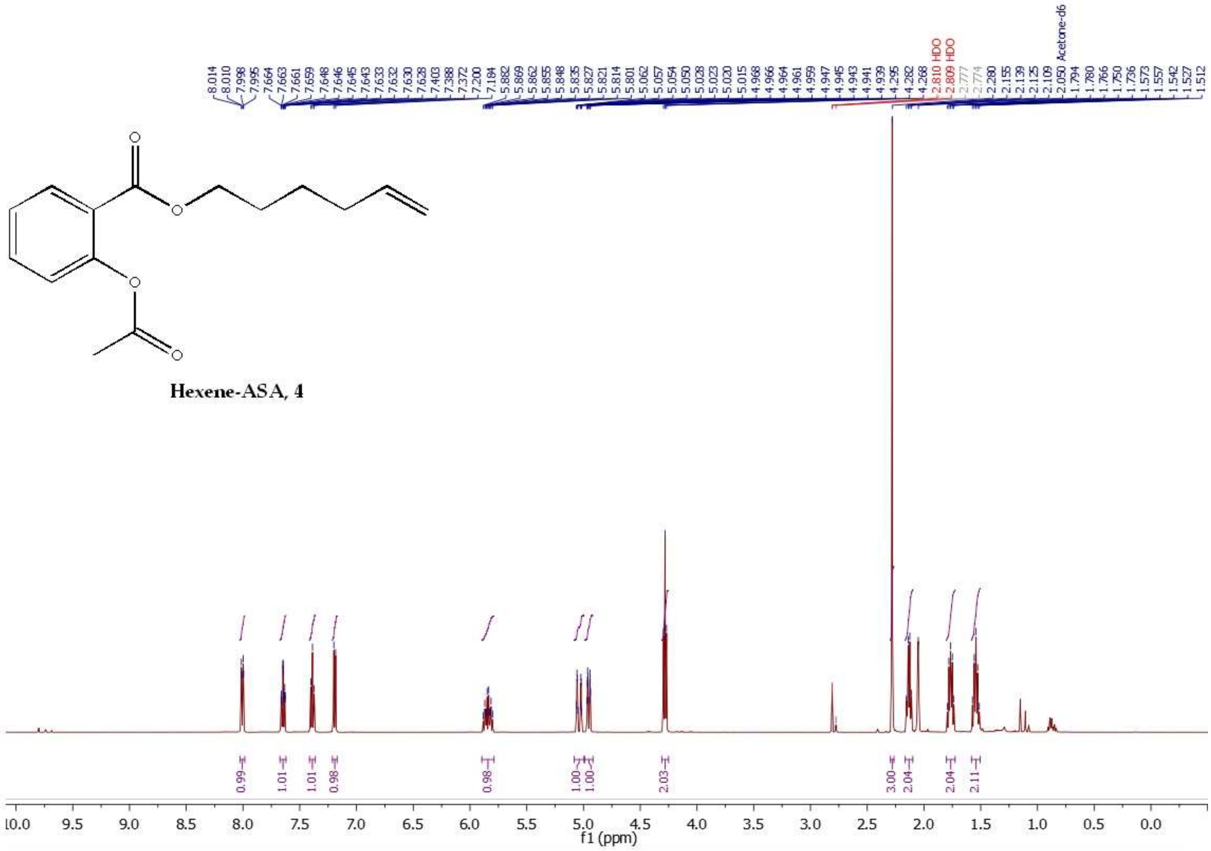

Figure S22. ^{1}H NMR spectrum of Hexene-ASA (**4**) in Acetone-*d6*

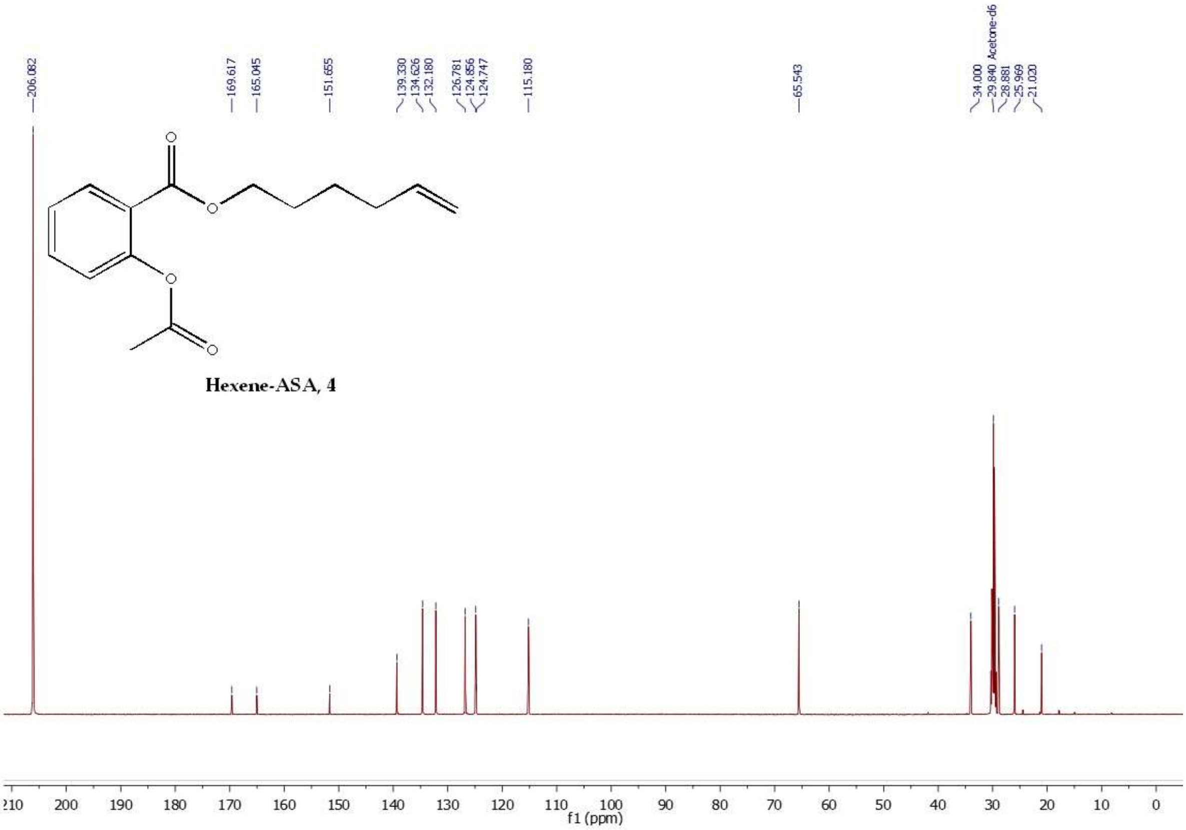

Figure S23. ^{13}C NMR of Hexene-ASA (**4**) in Acetone-*d6*

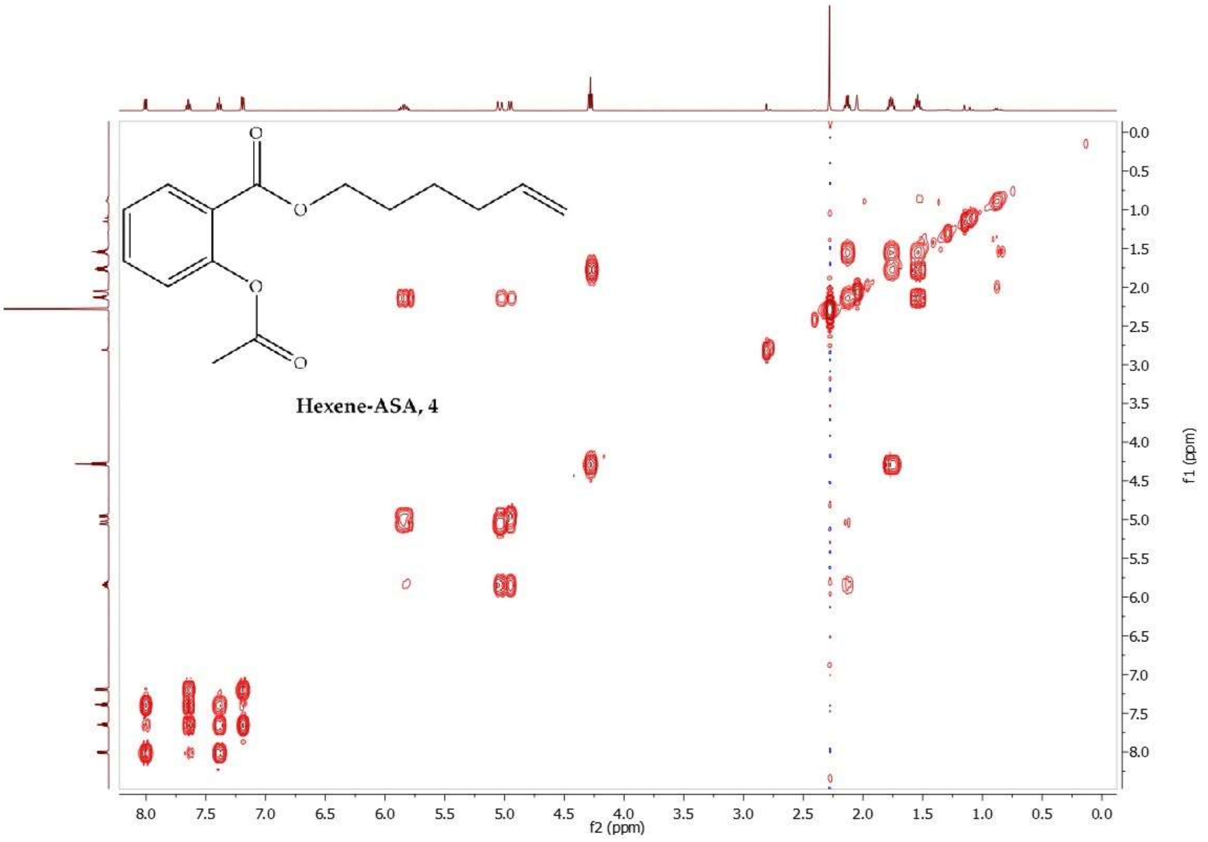

Figure S24. [^{1}H,^{1}H]-COSY of Hexene-ASA (**4**) in Acetone-*d6*

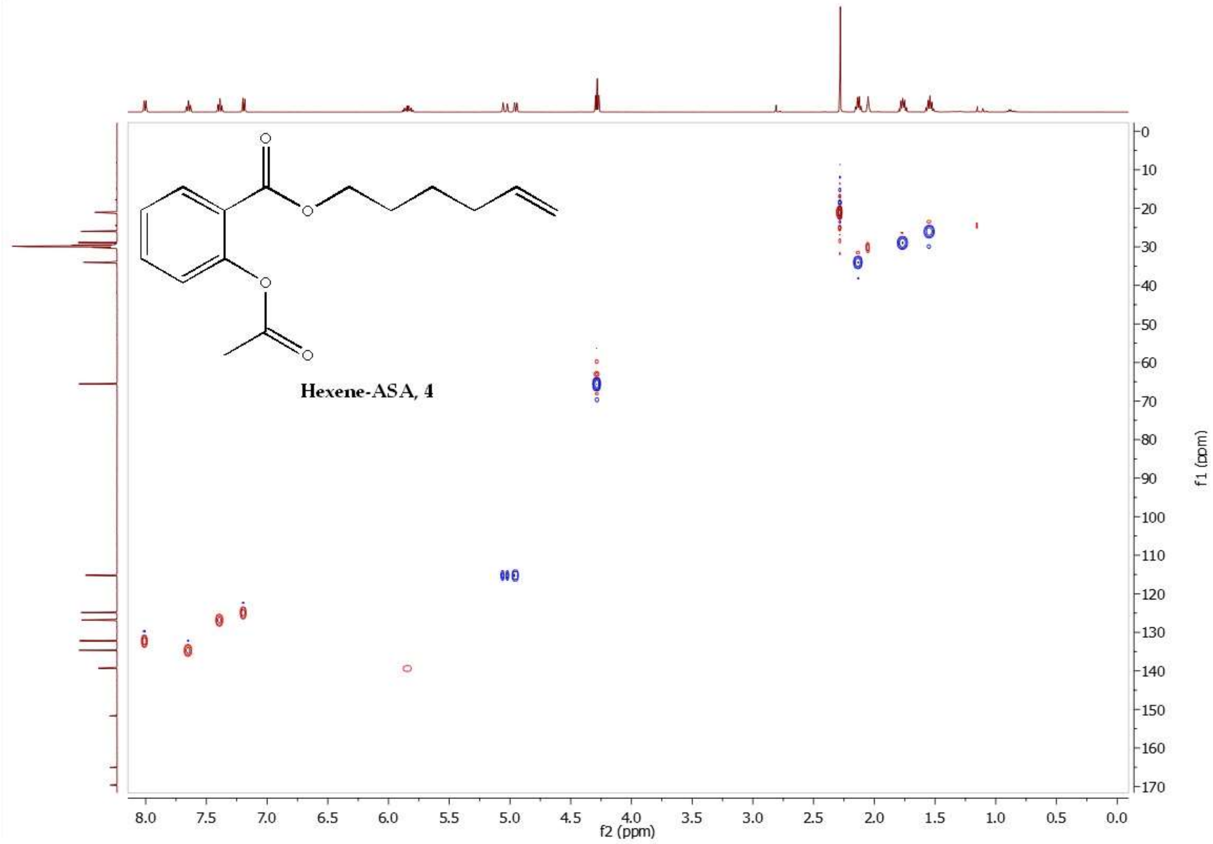

Figure S25. [^{1}H,^{13}C]-HSQC of Hexene-ASA (**4**) in Acetone-*d6*

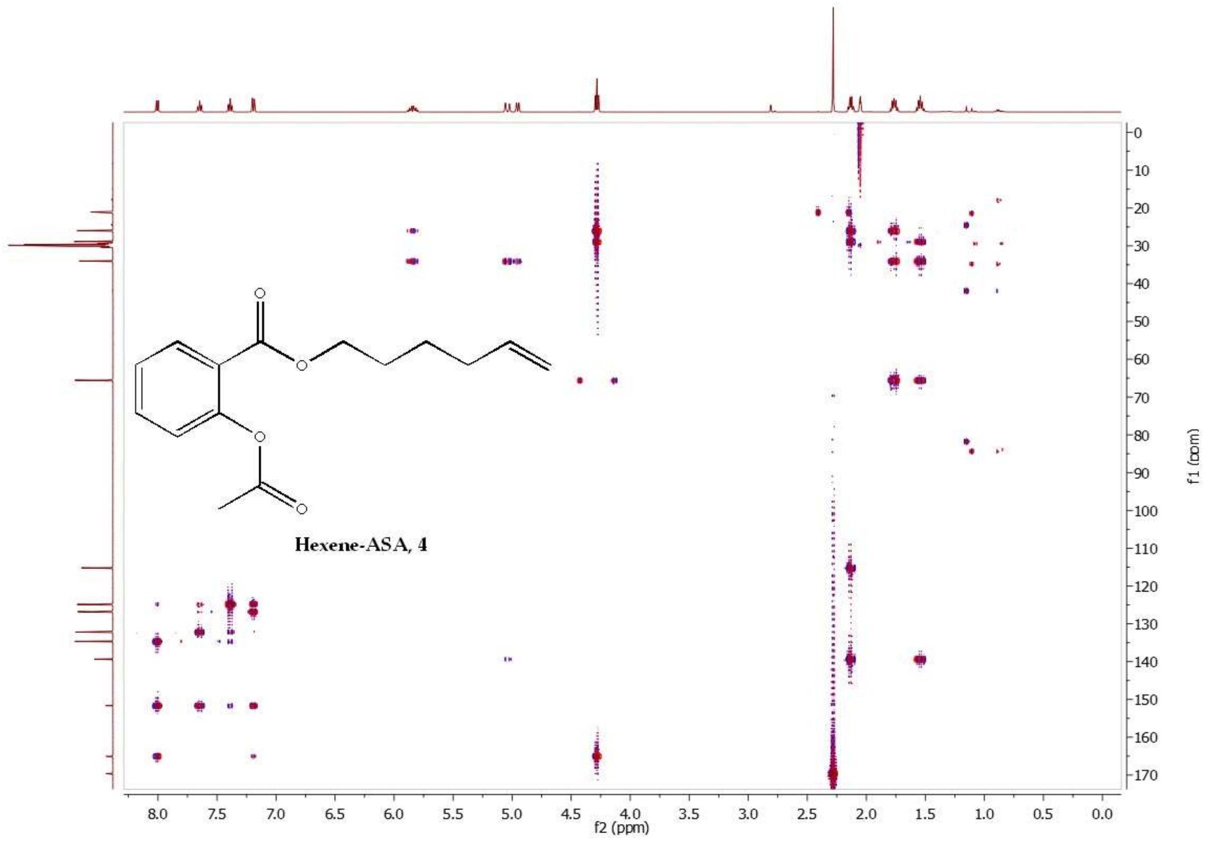

Figure S26. [^{1}H,^{13}C]-HMBC of Hexene-ASA (**4**) in Acetone-*d6*

2.8 Pt-Hexene-ASA (4a)

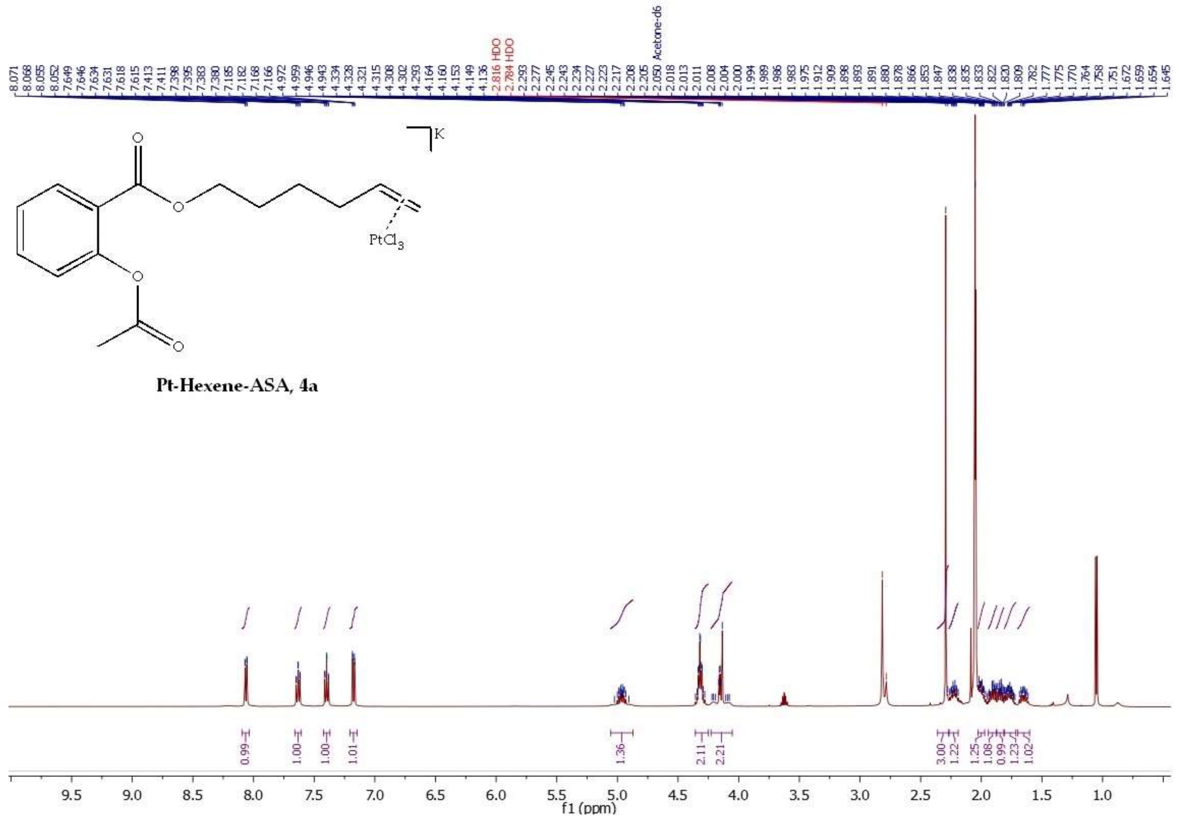

Figure S27. ^{1}H NMR of Pt-Hexene-ASA (**4a**) in Acetone-*d6*

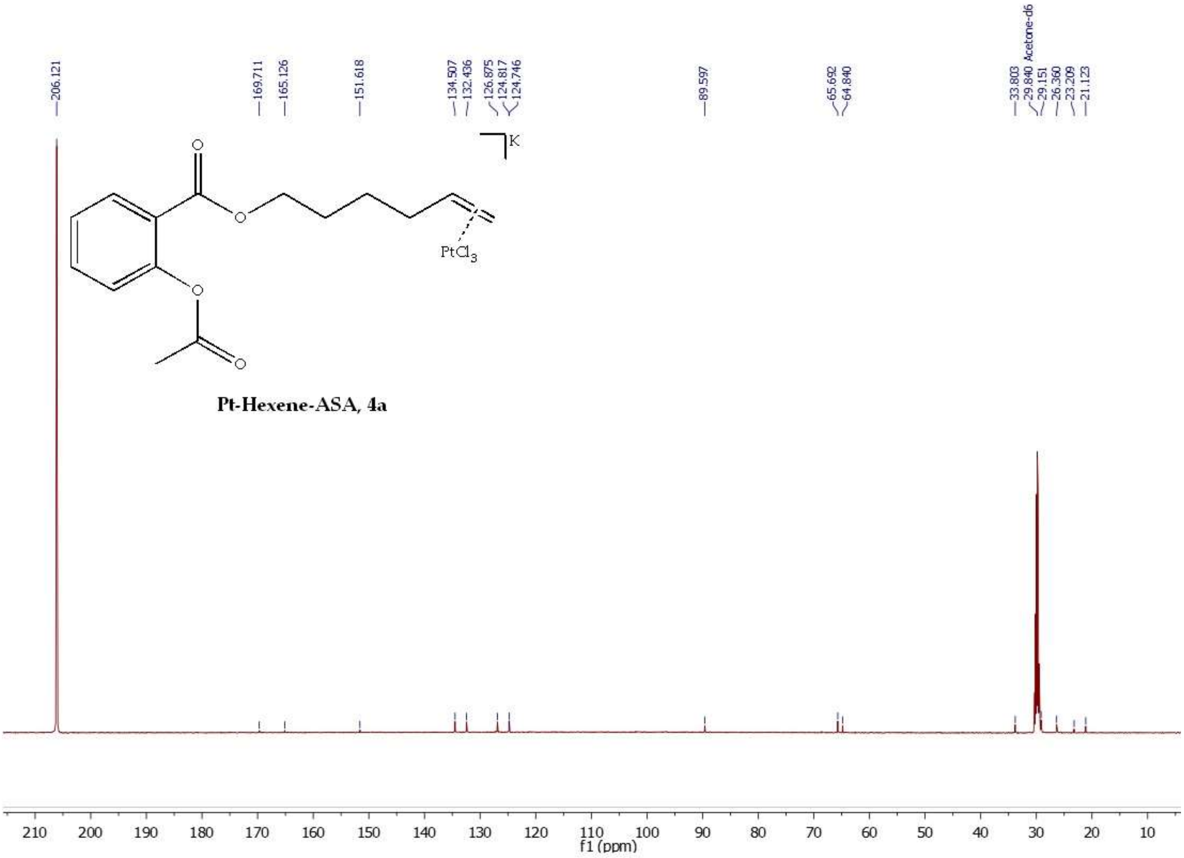

Figure S28. ^{13}C NMR of Pt-Hexene-ASA (**4a**) in Acetone-*d6*

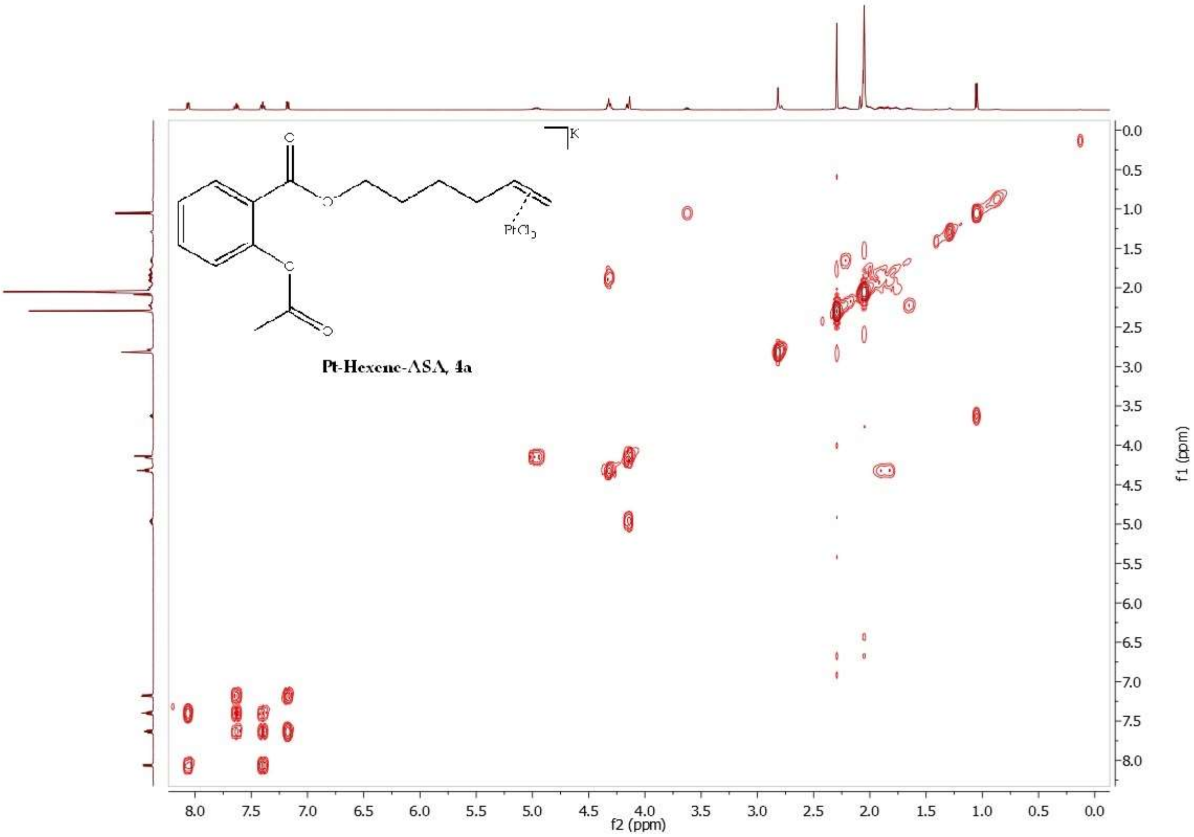

Figure S29. [^{1}H,^{1}H]-COSY of Pt-Hexene-ASA (**4a**) in Acetone-*d6*

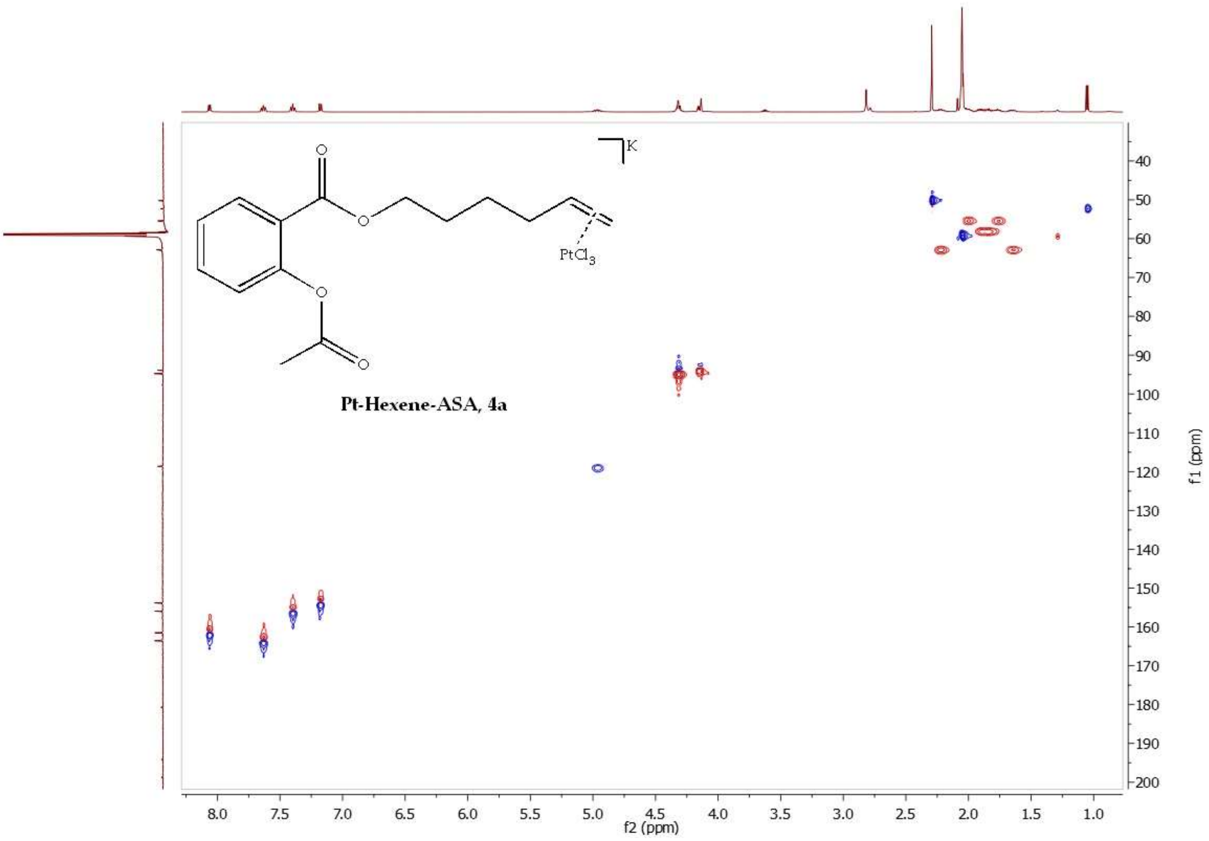

Figure S30. [^{1}H,^{13}C]-HSQC of Pt-Hexene-ASA (**4a**) in Acetone-*d6*

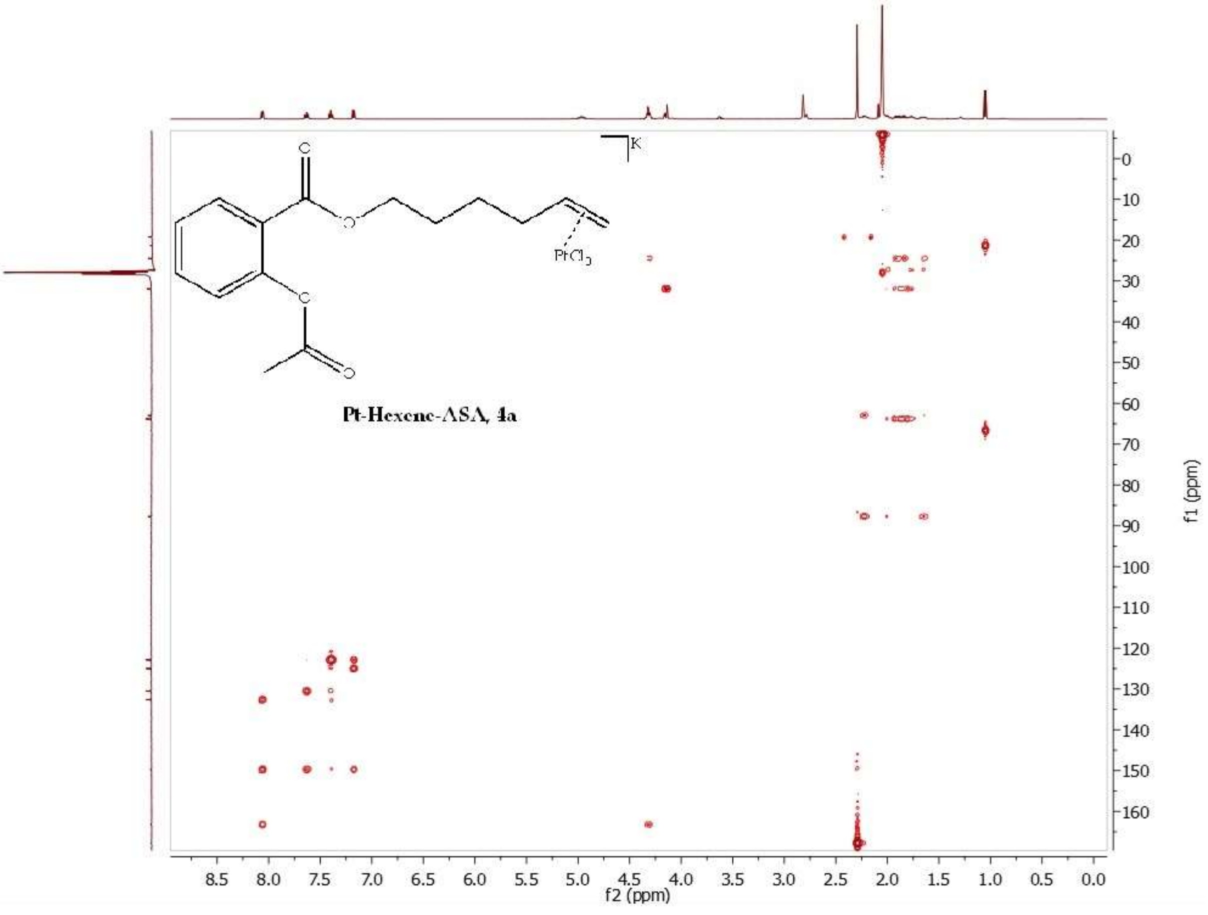

Figure S31. [^{1}H,^{13}C]-HMBC of Pt-Hexene-ASA (**4a**) in Acetone-*d6*

3. Crystal Data

Supplementary Table T1. Crystal Data and structure refinement for **1a**

Empirical formula	C16H22Cl3KO5Pt	
Formula weight	634.87	
Temperature	173(2) K	
Wavelength	0.71073 Å	
Crystal system	Monoclinic	
Space group	$P2_1/c$ (no. 14)	
Unit cell dimensions	a = 8.3367(3) Å	α = 90°
	b = 12.4677(5) Å	β = 92.7060(10)°
	c = 20.8667(9) Å	γ = 90°
Volume	2166.46(15) $Å^3$	
Z	4	
Density (calculated)	1.946 Mg/m^3	
Absorption coefficient	7.061 mm^{-1}	
F(000)	1224	
Crystal size	0.220 x 0.110 x 0.070 mm^3	
Theta range for data collection	2.446 to 25.997°	
Index ranges	-10<=h<=9, -15<=k<=15, -25<=l<=25	
Reflections collected	53983	
Independent reflections	4266 [R(int) = 0.0473]	
Completeness to theta = 25.242°	99.9%	
Absorption correction	Semi-empirical from equivalents	
Max. and min. transmission	0.862 and 0.598	
Refinement method	Full-matrix least-squares on F^2	
Data / restraints / parameters	4266 / 3 / 248	
Goodness-of-fit on F^2	1.089	
Final R indices [I>2sigma(I)]	R1 = 0.0186, wR2 = 0.0408	
R indices (all data)	R1 = 0.0234, wR2 = 0.0420	
Extinction coefficient	n/a	
Largest diff. peak and hole	0.946 and -1.446 e.$Å^{-3}$	
